# Geometry-invariant resonant cavities

**DOI:** 10.1038/ncomms10989

**Published:** 2016-03-24

**Authors:** I. Liberal, A. M. Mahmoud, N. Engheta

**Affiliations:** 1 Department of Electrical and Electronic Engineering, Universidad Pública de Navarra, E31006 Pamplona, Spain; 2 Department of Electrical and Systems Engineering, University of Pennsylvania, Philadelphia, Pennsylvania 19104, USA

## Abstract

Resonant cavities are one of the basic building blocks in various disciplines of science and technology, with numerous applications ranging from abstract theoretical modelling to everyday life devices. The eigenfrequencies of conventional cavities are a function of their geometry, and, thus, the size and shape of a resonant cavity is selected to operate at a specific frequency. Here we demonstrate theoretically the existence of geometry-invariant resonant cavities, that is, resonators whose eigenfrequencies are invariant with respect to geometrical deformations of their external boundaries. This effect is obtained by exploiting the unusual properties of zero-index metamaterials, such as epsilon-near-zero media, which enable decoupling of the temporal and spatial field variations in the lossless limit. This new class of resonators may inspire alternative design concepts, and it might lead to the first generation of deformable resonant devices.

## Abstract (web summary)

Resonant cavities are a ubiquitous building block in science and technology. Here, Liberal *et al*. demonstrate theoretically the existence of resonators whose eigenfrequencies are invariant with respect to geometrical deformations of their external boundaries, by exploiting the unusual properties of zero-index metamaterials.

## Graphical abstract

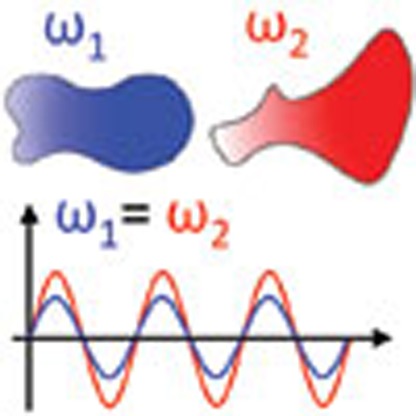

The dynamics of many physical systems are usually described in terms of wave equations subject to certain boundary conditions. This is the case, for example, in classical and quantum mechanics, electromagnetics, acoustics and fluid dynamics. Specifically, when considering source-free time-harmonic exp(−*iωt*) fields, one finds that the solutions to these equations, subject to specific boundary conditions, often take place at specific discrete *ω*-frequency values, usually labelled as eigenfrequencies, or resonance frequencies^1^. In general, wave equations interrelate both spatial and temporal variations of the fields (for example, consider the vector wave equation of the electric field in classical electromagnetics: 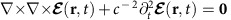 (ref. 2). Consequently, eigenfrequencies are determined by the geometry at hand.

This fundamental principle shapes the way we address various phenomena and develop technology. In fact, one of the main conceptual challenges that researchers, engineers and designers face across multiple disciplines is to come up with the appropriate geometry to operate at a specific frequency. On the other hand, fabrication imperfections degrade the performance of the devices, as well as hinder the application of thrilling physical concepts that may unfortunately require too stringent fabrication tolerances. Therefore, we could wonder if, as it is symbolically sketched in Fig. 1, it could be possible to find scenarios in which the eigenfrequencies of a resonant cavity are invariant with respect to geometrical transformations. If so, this would propose a complete change in the mindset behind design processes, and in turn open up the possibility for developing resonant devices that still be functional even under severe geometrical deformations with interesting applications, for example, in flexible photonics, as well as in tailoring light–matter interaction and quantum emission in such deformable structures.

Naturally, the idea of a geometry-invariant resonator challenges our intuition on how waves usually behave. However, the fields of topological insulators^3^,^4^ and topological photonics^5^,^6^,^7^,^8^,^9^,^10^ have revealed that certain physical quantities are preserved under continuous deformations. Moreover, during the past several years metamaterials have demonstrated that waves can be manipulated in unconventional manners^11^,^12^,^13^,^14^,^15^,^16^,^17^,^18^,^19^,^20^. For instance, metamaterials featuring extreme parameters, such as epsilon-and-mu-near-zero and zero refractive index structures, have been found to support fields with static spatial distributions, while maintaining their temporally dynamic properties^21^,^22^,^23^,^24^,^25^,^26^. This apparent decoupling between spatial and temporal domains encouraged us to believe that, indeed, resonators whose eigenfrequencies are invariant under geometrical transformations could be possible.

In the following, we will concentrate on the classical source-free time-harmonic wave equation for the electric field **E** in nonmagnetic media: 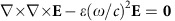 (ref. 2), with *c* being the speed of light in vacuum and *ɛ* the relative permittivity of the medium at hand. However, this must be considered only as a specific example of a more general concept that, as many other metamaterial paradigms, can be extrapolated to other forms of waves such as acoustic, elastic, mechanical and matter waves. Moreover, electromagnetic systems also represent an excellent test bench for future experimental verifications of the concepts introduced in this work. In fact, different experimental realizations of zero-index electromagnetic metamaterials have already been reported in the form of naturally available materials^27^,^28^, dispersion engineering in waveguides^29^,^30^, photonic crystals^25^ and artificial electromagnetic materials^31^,^32^.

In this work we analytically and numerically demonstrate that there are at least three distinct physical mechanisms in which zero-index metamaterials, and, in particular, epsilon-near-zero (ENZ) media, enable the development of cavities supporting eigenmodes whose eigenfrequency is invariant with respect to geometrical deformations of their external boundary.

## Results

### 2D cavities invariant under equi-areal transformations

One key property of ideal zero-index metamaterials is their ability to ‘stop' the spatial variations of the phase, and for some cases also the magnitude, of electromagnetic fields^21^,^22^,^23^,^24^. For instance, in ENZ media—that is, media whose relative permittivity is approximately zero, *ɛ*≈0—the magnetic field parallel with the axis of a two-dimensional (2D) system must be uniform, 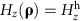, to avoid a singularity of the electric field 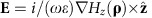 (refs 22, 23, 24). One could anticipate that the influence of geometry is lessened in the presence of spatially uniform fields, since effectively the apparent wavelength in such media is very large. This intuition is indeed correct, and we show that uniform magnetic field distributions can be associated with 2D cavities whose eigenfrequency is invariant with respect to equi-areal transformations.

**Figure 2:**
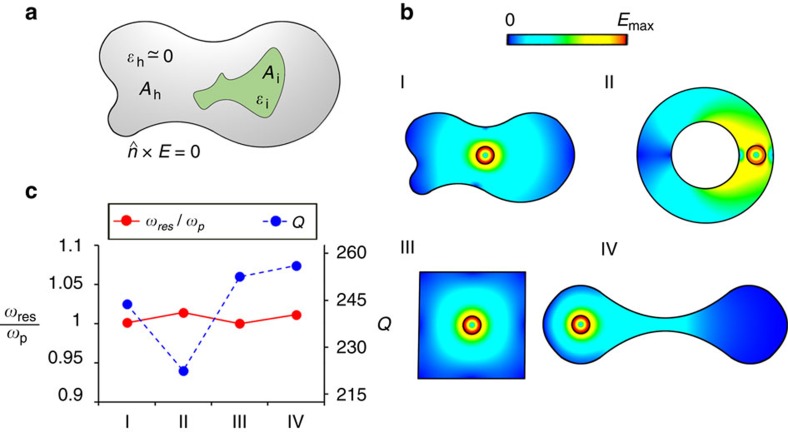
Two-dimensional (2D) cavities with eigenfrequencies invariant under equi-areal geometry deformations. (**a**) Sketch of a generic 2D cavity composed of a 2D ENZ host of cross-sectional area *A*_h_ containing a 2D dielectric (nonmagnetic) particle of cross-sectional area *A*_i_, perimeter *L*_i_ and relative permittivity 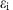. The entire 2D cavity is bounded by a PEC wall on which the tangential component of **E** field must vanish. (**b**) Colourmaps of the electric field magnitude distributions of resonant eigenmodes, obtained using numerical simulation, in four cavities consisting of a Si (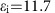) cylinder of radius *r*_i_=1.165 μm, immersed in a 2D SiC host of different shapes but equal cross-sectional area 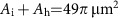. (**c**) Linear graph portraying the resonance frequency (normalized to the SiC plasma frequency) and the quality factor *Q* as a function of cavity number. Here the imaginary part of relative permittivity of SiC is assumed to be 0.1 at the SiC plasma frequency. In Supplementary Fig. 5, we show how these quantities vary with different level of loss (as represented by the different value of the imaginary part of permittivity of SiC).

To this end, let us consider, for example, a 2D cavity composed of a 2D dielectric particle of relative permittivity *ɛ*_i_, cross-sectional area *A*_i_ and perimeter *L*_i_, immersed in a 2D ENZ host of arbitrary cross-sectional shape but area *A*_h_ (Fig. 2a), bounded by perfectly electric conducting (PEC) walls. As demonstrated in Supplementary Note 1, the eigenfrequencies obtained as solutions to the source-free electric field time-harmonic wave equation subject to the boundary condition 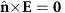 on the PEC wall are determined by the solutions to the following characteristic equation: 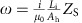, where 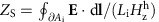 is the surface impedance of the particle embedded in ENZ. It is thus clear that the existence of an eigenmode at the ENZ frequency is completely determined by the overall cross-sectional area of the ENZ host, *A*_h_, and the properties of the internal 2D particle, encapsulated in *Z*_S_. Therefore, if the internal particle is designed so that the characteristic equation has a solution at the plasma frequency of the host, then the cavity will have an eigenmode at such eigenfrequency, independently of the shape of its external boundary, as long as its cross-sectional area remains the same. As if it were an incompressible fluid, the 2D cavity can be exposed to any equi-areal geometrical deformation while keeping the same eigenfrequency. Note that the set of allowed geometrical deformations also includes piercing (making 2D holes in) the cavity. For example, a simply connected cavity can be transformed into a 2D annulus-like cavity. If the total area as well as the geometry and the material of the internal 2D particle are kept the same, the eigenfrequency will be immune even to evident changes in the topology of the cavity.

To illustrate this fact, we can, for example, set the internal particle as an infinitely long cylinder of relative permittivity *ɛ*_i_ and radius *r*_i_. In such a case, the surface impedance of the particle can be written in closed form: 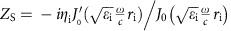, where 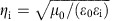 is the intrinsic medium impedance in the particle, *J*_0_(*x*) is the cylindrical Bessel function of the first kind and order zero, and 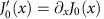. Consequently, the characteristic equation can be explicitly written as follows: 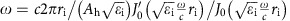, where it is again evident that the existence of an eigenmode with uniform magnetic field in the ENZ host medium only depends on the characteristics of the internal particle (*ɛ*_i_ and *r*_i_), and the cross-sectional area of the ENZ host *A*_h_. Therefore, if the radius of the cylinder is set so that the characteristic equation has a solution at the plasma frequency of the host, then the cavity will have an eigenmode whose eigenfrequency is independent with respect to equi-areal transformations of the ENZ host.

To validate this property, we have numerically studied a few examples of 2D cavities by carrying out an eigenfrequency analysis with a full-wave electromagnetic solver (Fig. 2b). These specific examples have been selected to illustrate the high degree of arbitrariness in the geometry of the 2D cavities, including non-canonical shapes (Fig. 2b, I), different topologies (Fig. 2b, II), sharp corners (Fig. 2b, III) and high-aspect ratios (Fig. 2b, IV). A more detailed description of the geometry of these cavities is gathered in Supplementary Figs 1–4. Anticipating future experimental verifications of the presented results, the ENZ host has been modelled using silicon carbide (SiC)^27^, whereas the internal dielectric cylinder is assumed to be silicon (Si), with relative permittivity *ɛ*_i_=11.7 (ref. 33). In this manner, our analysis includes the effect of the relatively high losses of SiC with relative value of the imaginary part of its permittivity to be 0.1 (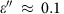) in the vicinity of the SiC plasma frequency, 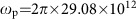 rad s^−1^ (corresponding to a free-space wavelength of around 10.3 μm), where the real part of the relative permittivity is near zero (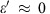)^27^. The radius of the cylinder (*r*_i_=1.165 μm) and the area of the host (*A*_h_=49*π* μm^2^) have been selected such that the characteristic equation is satisfied at the SiC plasma frequency using a cylinder that is subwavelength in its cross-section.

The eigenfrequencies of these 2D resonators were computed numerically and are depicted in Fig. 2c. It is apparent from the figure that despite their very distinct geometry, and despite the fact that realistic losses have been taken into account, the eigenfrequencies of these resonators deviate by <1.5% from the plasma frequency of SiC. These small disagreements are mainly caused by the losses of SiC, which slightly deviate the response of the host from that of a pure ENZ medium. As a matter of fact, the eigenfrequencies converge even more closely to the SiC plasma frequency as losses decrease (Supplementary Fig. 5).

It is worth remarking that, unlike its eigenfrequency, not all properties of the resonator are invariant with respect to geometrical deformations. For example, the quality factor *Q*—i.e., the ratio between the stored and dissipated energies per cycle—strongly depends on the field intensity distributions in the resonator^2^. Subsequently, as it is illustrated in Fig. 2c, deforming the cavities results in changes in the quality factor in an excess of 10%, while their resonance frequencies stay effectively unchanged. This exotic feature could be potentially exploited, for instance, in designing flexible resonators in which the strength of the coupling with a quantum emitter embedded in them can be modified by deforming their external boundary, while keeping a constant resonance frequency, hence enabling a fine tuning of the decay dynamics of the quantum emitter. This is the subject of our ongoing study and will be reported in a future publication.

Moreover, Fig. 2c and Supplementary Fig. 5 also serve to illustrate a unique property of the proposed geometry-invariant resonators that, to the best of our knowledge, has no counterpart in conventional resonators. Specifically, in a conventional resonator, the quality factor increases as losses decrease, and the eigenfrequency becomes more sensitive to geometrical deformations. On the contrary, in our proposed geometry-invariant resonators, the smaller the losses the larger the quality factor, but also the more robust the eigenfrequency is towards geometrical deformations (Supplementary Fig. 5). In this manner, the proposed idea in principle enables the development of high *Q* resonators whose eigenfrequencies are immune to geometrical deformations.

To finalize the discussion for this set of modes, we emphasize that the choice of a 2D cylindrical internal particle with circular cross-section was made for the sake of simplicity, and to have an analytical solution to the characteristic equation, hence facilitating the comparison between theory and numerical simulations. However, in principle any other 2D particle could be utilized to induce an eigenmode in the 2D ENZ host. For example, an analogous analysis by using a 2D particle with square cross-section is reported in Supplementary Figs 6 and 7, leading to the same conclusions.

### 3D cavities supporting spatially ‘electrostatic' eigenmodes

Next, even a more general invariance with respect to geometrical deformations may be found by noting that, as demonstrated in Supplementary Note 2, ENZ media may also support other modes as exp(−*iωt*) time-varying spatially ‘electrostatic' fields, which are different from what we discussed above. In other words, as the medium relative permittivity goes to zero, the Maxwell curl equation 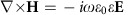 may also support solutions with zero magnetic field **H**=**0**, but a non-zero and time-varying electric field, 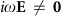. Naturally, the other Maxwell curl equation imposes that the associated electric field is irrotational 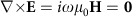, since for this mode **H** is zero in the ENZ region. Thus, interestingly, ENZ media may support solutions to the wave equation in the form of spatially ‘electrostatic' distributions that are dynamically varying in time. We note that the existence of time-varying electrostatic field distributions had already been discovered in the field of plasma physics, mostly in the form of longitudinal waves^34^. Here we remark that ENZ media support generic electrostatic field distributions, which can be excited in a wide set of cavities.

The field distributions of these spatially electrostatic eigenmodes correspond to the solution of Laplace's equation (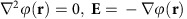) in the ENZ host, subject to the appropriate boundary conditions. Interestingly, the solution to the Dirichlet problem of Laplace's equation is known to exist and be unique if the boundary is sufficiently smooth and the potential prescribed at the boundary is continuous^35^. Therefore, if the boundary conditions on the ENZ host enable the existence of spatially electrostatic modes, then such a cavity has an eigenfrequency at the ENZ frequency, no matter what its geometry is.

To illustrate this phenomenon with a specific example, let us consider a three-dimensional (3D) scenario in which a resonator is composed by a 3D dielectric particle immersed in a 3D ENZ host. A detailed theoretical derivation of the conditions under which this composite cavity supports an eigenmode is included in Supplementary Note 3 and Supplementary Fig. 8. However, it is actually sufficient to simply note that to excite a spatially ‘electrostatic' eigenmode with zero magnetic field in the ENZ region, the continuity of the fields imposes that the magnetic field (normal and tangential) at the boundary of the dielectric particle must be zero. In addition, at this boundary the normal component of the electric field inside the dielectric particle must also be zero to preserve the continuity of the normal displacement vector. (Note that the electric field inside the ENZ host might have a normal component to this boundary of dielectric particle, still satisfying the continuity of the normal displacement vector.) If we find a particle satisfying these conditions, then the composite cavity particle plus ENZ host will support an eigenmode at the plasma frequency, no matter what the geometry of the ENZ host is.

For example, if the internal particle is a dielectric sphere of radius *r*_i_ and relative permittivity *ɛ*_i_, then these conditions are met at the solutions of the following characteristic equation: 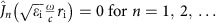 (see also Supplementary Notes 3 and 4, as well as Supplementary Fig. 9 for the analysis of a canonical core-shell cavity). That is to say, the eigenfrequencies of the resonator correspond to the zeros of the functions 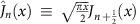, representing the Schelkunoff form of the spherical Bessel functions of the first kind and order *n*, where *J*_*n*_(*x*) is the cylindrical Bessel function of the first kind and order *n* (ref. 2). Note that in this case there is not only one, but an infinite number of possible eigenmodes *n*=1, 2, 3… with geometry-invariant properties. Moreover, due to the spherical symmetry of the internal particle, there are 2*n*+1 degenerate modes for each *n*-th eigenmode. We emphasize that the solutions to this characteristic equation only depend on the properties on the internal particle 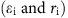, and are independent of the geometry of the main cavity. Therefore, once the internal particle has been correctly designed, then the ideal ENZ host, and hence the external boundaries of the cavity, can in principle be of any size and shape. What is more, the cavity could even be ‘polluted' with other particles made of different dielectric materials sharing the same ENZ host medium. In all these cases, the cavity will support an eigenmode at the ENZ frequency. As shown in Supplementary Note 5 and Supplementary Fig. 10, the invariance of the eigenfrequency in the presence of time-harmonic spatially electrostatic fields can also be justified by using perturbational techniques. These modes can be excited in both 2D and 3D systems.

**Figure 3:**
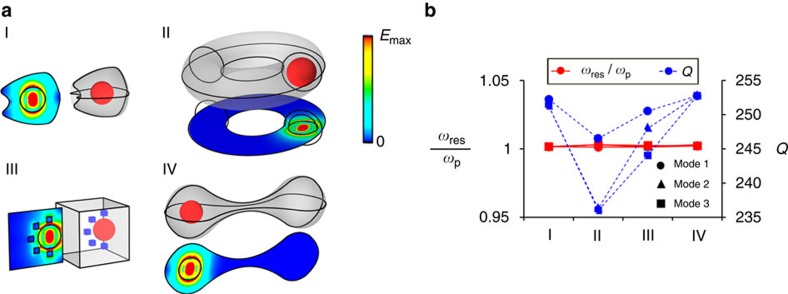
Three-dimensional (3D) cavities supporting spatially ‘electrostatic' modes with the same resonance eigenfrequency. (**a**) Four 3D cavities with different geometries supporting the same resonance eigenfrequency. Each cavity consists of a Si sphere (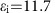) (shown as a red sphere) of radius 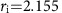 μm immersed in a 3D SiC host (shown as grey background), bounded by a PEC wall. Cavity III also contains several additional cubic dielectric particles (shown in blue) with permittivity 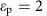, and side 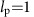 μm inserted in the ENZ host. Next to each we show the colourmaps of the electric field magnitude distributions obtained using numerical simulation of one of the three supported degenerate eigenmodes (the other eigenmodes can be found in Supplementary Figs 15–18). (**b**) Linear graph portraying the resonance frequency (normalized to the SiC plasma frequency) and quality factors for these four cavities, demonstrating that the resonance eigenfrequencies are the same, while the quality factors of these eigenmodes are different. Here the imaginary part of relative permittivity of SiC is assumed to be 0.1 at the SiC plasma frequency.

The geometry-invariant properties of these eigenmodes are numerically validated in Fig. 3a, which shows four cavities with very distinct geometries, but that nevertheless support eigenmodes at the same eigenfrequency. Again, these specific cavities have been chosen to illustrate the high degree of arbitrariness in the geometry of the cavities (shape, topology and in this case also size). A more detailed description of their geometry can be found in Supplementary Figs 11–14. All cavities are composed of a SiC host containing a Si particle. In this case, a spherical particle of radius 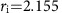 μm has been selected to satisfy the characteristic equation, 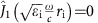 (that is, 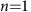), at the SiC plasma frequency. For the sake of brevity, Fig. 3a only depicts the electric field magnitude distribution of one of the three degenerate modes that can be excited in the vicinity of the SiC plasma frequency (each eigenmode corresponding to a different orientation of the electric dipolar mode within the Si spherical particle). The electric and magnetic field magnitude distributions of all degenerate modes are depicted in Supplementary Figs 15–18. The fact that the magnetic field vanishes in the ENZ host can also be more clearly appreciated in those figures. The resonance frequencies and quality factors of these modes have been numerically computed and are depicted in Fig. 3b. Despite the use of the realistic losses of SiC 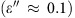, the numerical computation of the resonance frequencies reveals that all degenerate modes in all four cavities deviate <0.3% from the SiC plasma frequency. Furthermore, the fact that degenerate modes exhibit different quality factors allows us to envision the design of a new class of resonators, in which the fields excited by quantum emitters immersed within them exhibit a different Purcell factor and decay dynamics as a function of their polarization, while maintaining the same resonance frequency.

Again, we remark that while the internal particle must be designed to enable the excitation of an eigemode at the ENZ frequency, this particle must not necessarily be the sphere used in the current example. In essence, any particle supporting a solution to the wave equation in which the magnetic field and the normal electric field are zero at its boundary can trigger the excitation of a spatially ‘electrostatic' mode in an arbitrarily shaped ENZ region. For instance, Supplementary Figs 19–23 present an equivalent analysis for the case in which the cavities contain a cylindrical dielectric particle whose top and bottom walls have been covered by perfect magnetic conductor layers. The results are very similar to those obtained in Fig. 3.

### Surface-avoiding modes

To finalize, there is at least a third set of modes present in ENZ media that are invariant under certain (but not completely arbitrary) geometrical transformations. These modes correspond to the cases where both electric and magnetic fields are neither constant nor zero in the ENZ region. Note that, even if not constant, the magnetic field must always be irrotational 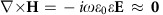, and thus it features a ‘quasi-static' spatial distribution while it is temporally dynamic. However, in this case, the electric field cannot be curl free,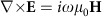, and it indeed takes the form of a solenoidal field forming closed loops in the cavity. The geometry-invariant properties of this set of modes arise from the fact that the modes can concentrate the fields on the vicinity of the internal dielectric particle, resulting in a negligible field at the outer boundaries of the cavity, which naturally satisfies the 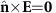 boundary condition. In essence, the ENZ properties of the medium ensure that the propagation constant vanishes, 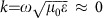, and, hence, the fields cannot propagate as in a conventional dielectric through the ENZ host towards the external surface of the cavity.

**Figure 4:**
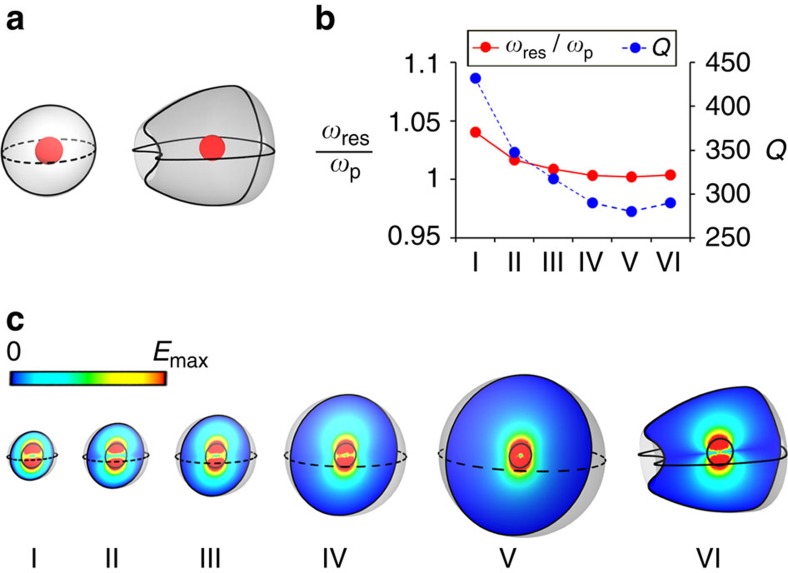
Three-dimensional (3D) cavities supporting surface-avoiding modes. (**a**) Sketches of two cavities consisting of a Si sphere (shown as a red sphere) with 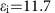 and radius 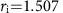 μm immersed in a 3D SiC host (shown as a grey background). (**b**) Linear graph portraying the resonance frequency (normalized to the SiC plasma frequency) and quality factor for six cavities, showing small variation in the resonance frequency, while having different quality factors. Here the imaginary part of relative permittivity of SiC is assumed to be 0.1 at the SiC plasma frequency. (**c**) Colourmaps of the electric field magnitude distributions of the surface-avoiding eigenmodes, obtained using numerical simulations, in six different cavities (five of which are obtained from the left cavity but with different sizes of the ENZ host, whereas the sixth is the second cavity, shown in the top left panel).

For instance, let us consider a spherical cavity with two concentric layers, as schematically depicted in Fig. 4a. In particular, we assume that the inner layer (the internal particle) is made of Si, whereas the external layer (the background host) is filled with SiC. In this manner, when the radius of the internal particle, *r*_i_, is much smaller than the external radius of the cavity, *r*_out_, that is, 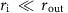, the field on the surface of the cavity is negligible, and the eigenfrequency becomes approximately independent of the volume and shape of the resonator. Specifically, and as shown in Supplementary Note 4, the characteristic equation determining the eigenfrequencies of this set of modes in our spherical example can be asymptotically written as follows: 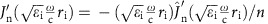, for 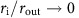, 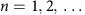. Fig. 4 gathers a set of examples illustrating how, as the volume of the cavity increases, the eigenfrequencies converge towards the value prescribed by the characteristic equation. It also exemplifies that once the cavity is sufficiently large, the eigenfrequency becomes independent of the shape of the external surface of the cavity. The examples have been chosen to illustrate the impact of progressively increasing the size, and the geometry of the cavities is detailed in Supplementary Figs 24 and 25. In this case, the radius of the internal particle (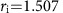 μm) is selected so that the eigenfrequency satisfying the characteristic equation for *n*=1 equals the SiC plasma frequency.

## Discussion

In summary, our theoretical study demonstrates that in the context of ENZ and zero-index metamaterials there are multiple solutions to the wave equation whose eigenfrequency is invariant under geometrical transformations. It was demonstrated that these solutions enable the design of resonant cavities whose eigenfrequencies are invariant with respect to geometrical transformations of their external boundary, and, hence, they inspire new design philosophies in which the geometry of a device is not determined by, and locked to, its frequency of operation. While our analytical and numerical analyses have been focused on closed cavities bounded by PEC walls, we expect that analogous phenomena could be observed in open resonators. A set of preliminary simulations is included in Supplementary Figs 26–31. We believe that these unconventional resonators could give rise to a new generation of deformable resonant devices. Among other applications, the proposed resonators appear to be particularly well suited for flexible photonics and cavity quantum electrodynamics. For instance, the proposed cavities can be locked with their resonances overlapping the atomic transitions of quantum emitters embedded within them, while different aspects of the emitter-cavity interaction can be dynamically tuned by means of deforming the cavity.

## Methods

### Numerical simulations

The commercially available full-wave electromagnetic simulator software COMSOL Multiphysics, version 5.0, was used to generate all 2D and 3D numerical simulations presented in the figures of the main text and the Supplementary Material. Specifically, we carried out eigenfrequency analyses in which the software makes use of the finite element method to determine the eigenmodes and eigenfrequencies of the wave equation: 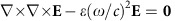, subject to be PEC boundary condition, that is, the tangential electric field vanishes on the external surface of the cavity, 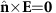, where 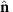 stands for the outward normal vector to the surface of the cavity. The solver was requested to search for eigenfrequencies around the frequency where the real part of the permittivity is near zero 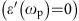, and the eigenmodes with the closest eigenfrequencies to such a frequency were selected. The ENZ host was modelled as SiC in accordance with ref. 27. Thus, the plasma frequency, at which the real part of the permittivity vanishes, 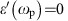, takes place at *ω*_p_=2*π* × 29.08 10^12^ rad s^−1^, with losses represented by 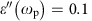 unless otherwise stated. The numerical solver provided the field distributions of the eigenmodes and their associated eigenfrequencies. These data were also used to calculate the quality factor *Q* as the ratio of the energies stored and dissipated per unit cycle: 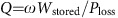, where 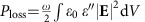 and 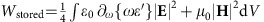 were computed via integration of the electric and magnetic field intensities. In Supplementary Figs 26–31 the analysis was carried out without any PEC boundary and using the frequency domain solver. The simulation set-up is described in Supplementary Figs 26 and 29.

## Additional information

**How to cite this article:** Liberal, I. *et al*. Geometry-invariant resonant cavities. *Nat. Commun.* 7:10989 doi: 10.1038/ncomms10989 (2016).

## Supplementary Material

Supplementary Information Supplementary Figures 1-31, Supplementary Notes 1-5 and Supplementary References.

## Figures and Tables

**Figure 1:**
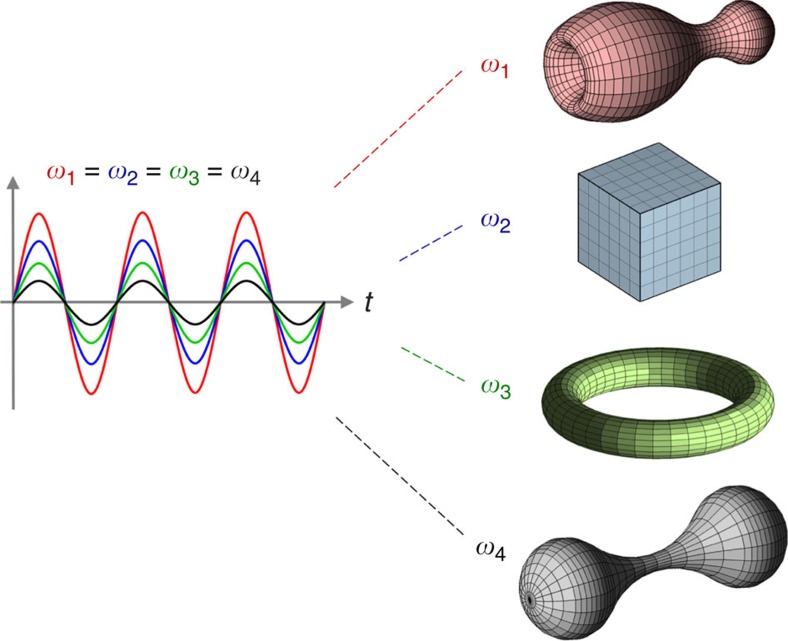
Geometry-invariant resonant cavities. Conceptual sketch of resonant cavities that, despite their very distinct geometry (shape, size, topology), support an eigenmode at the same resonance frequency.
