# Supplementary material for: Geometry-invariant resonant cavities
Source: Nat Commun. 2016 Mar 24;7:10989. doi: 10.1038/ncomms10989 (PMC4820806; doi:10.1038/ncomms10989)
Supplement: Supplementary Information — Supplementary Figures 1-31, Supplementary Notes 1-5 and Supplementary References. [file ncomms10989-s1.pdf]

Supplementary Figures:

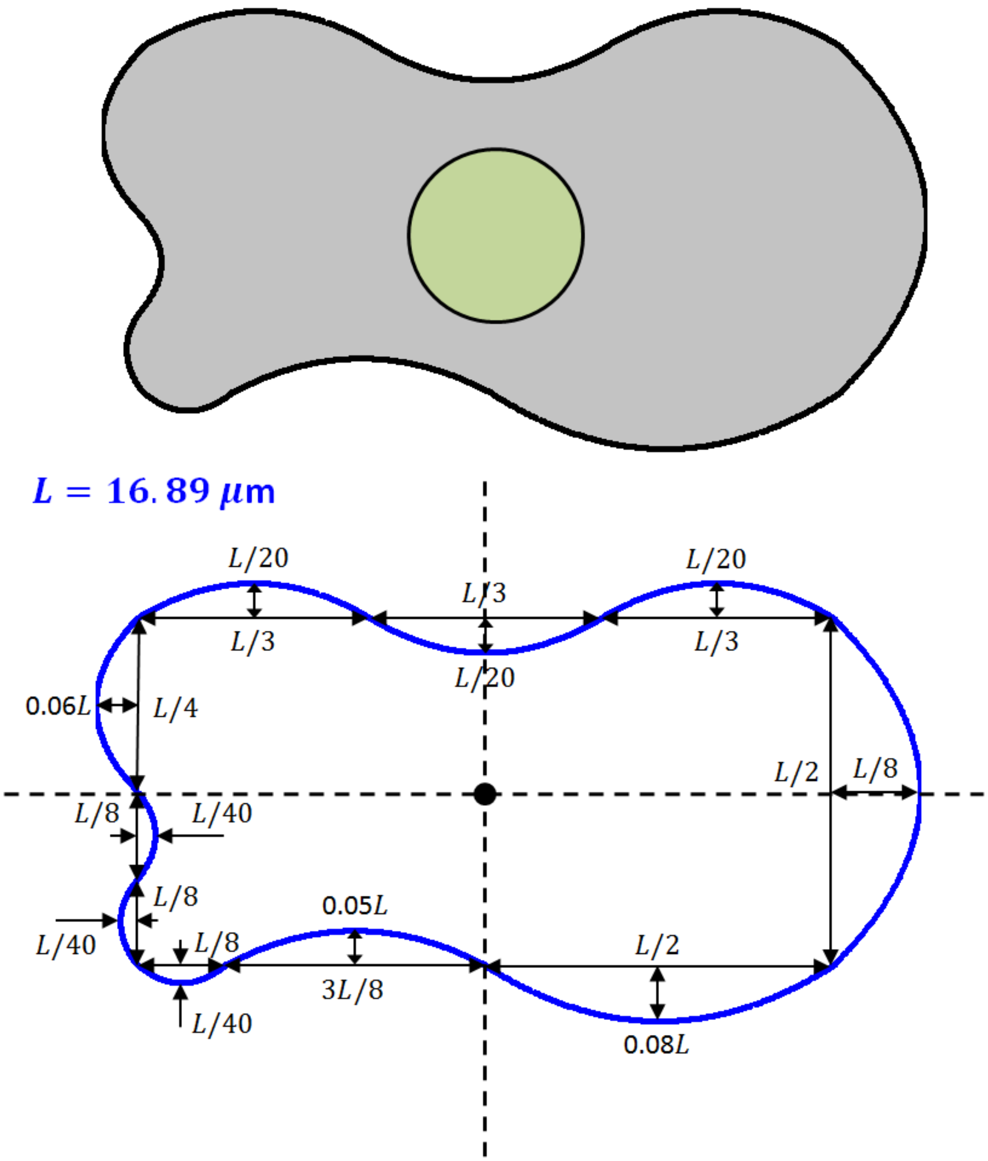

Supplementary Figure 1: Sketch (top) and dimensions (bottom) of the cavity studied in Fig. 2B-I of the main text. The cavity consists of a Si cylinder ($\varepsilon_i = 11.7$, shown in green) of radius $r_i = 1.165$ µm immersed in a SiC host (shown as grey background). The location of the sphere in the main cavity is indicated by a black dot. The blue curves correspond to the second order polynomials $f(u) = c_0 + c_1 u + c_2 u^2$ that fit to the specified dimensions. The resulting area is $\pi 7^2\ \mu$m.

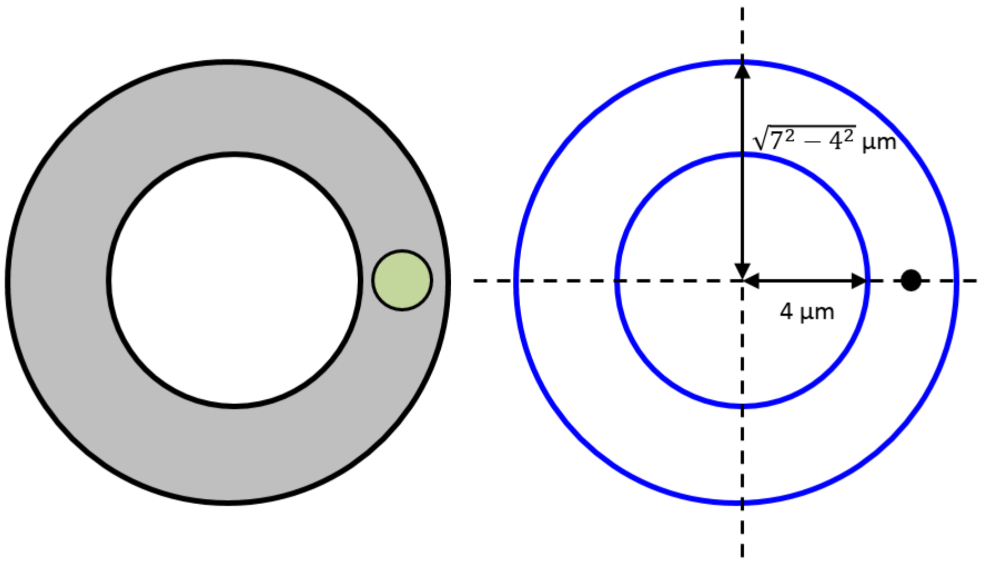

Supplementary Figure 2: Sketch (left) and dimensions (right) of the cavity studied in Fig. 2B-II of the main text. The cavity consists of a Si cylinder ($\varepsilon_i = 11.7$) (shown in green) of radius $r_i = 1.165$ µm immersed in a SiC host (shown as grey background). The location of the sphere in the main cavity is indicated by a black dot. The area contained between the two blue circles conforming the main cavity also equals $\pi 7^2$ μm.

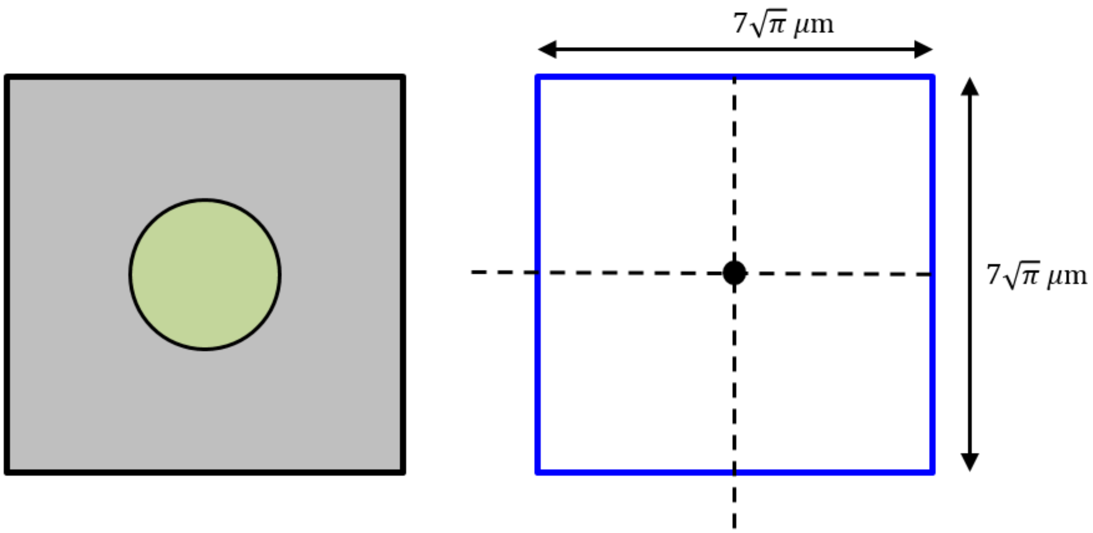

Supplementary Figure 3: Sketch (left) and dimensions (right) of the cavity studied in Fig. 2B-III of the main text. The cavity consists of a Si cylinder ($\varepsilon_i = 11.7$) (shown in green) of radius $r_i = 1.165$ µm immersed in a SiC host (shown as grey background). The location of the sphere in the main cavity is indicated by a black dot. The area of the main cavity also equals $\pi 7^2$ μm.

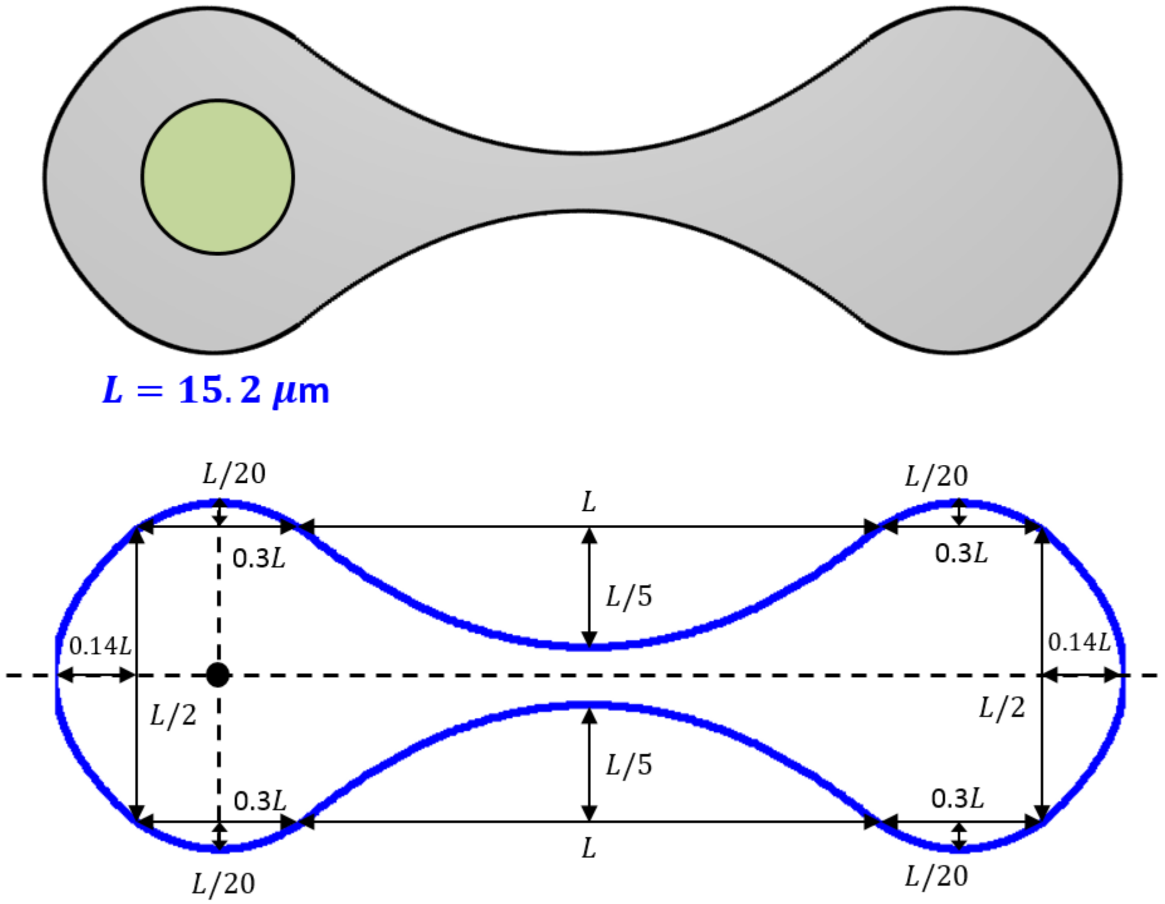

Supplementary Figure 4: Sketch (top) and dimensions (bottom) of the cavity studied in Fig. 2B-IV of the main text. The cavity consists of a Si cylinder ($\varepsilon_i = 11.7$) (shown in green) of radius $r_i = 1.165$ µm immersed in a SiC host (shown as grey background). The location of the sphere in the main cavity is indicated by a black dot. The blue curves correspond to the second order polynomials $f(u) = c_0 + c_1 u + c_2 u^2$ that fit to the specified dimensions. The resulting area is also $\pi 7^2$ μm.

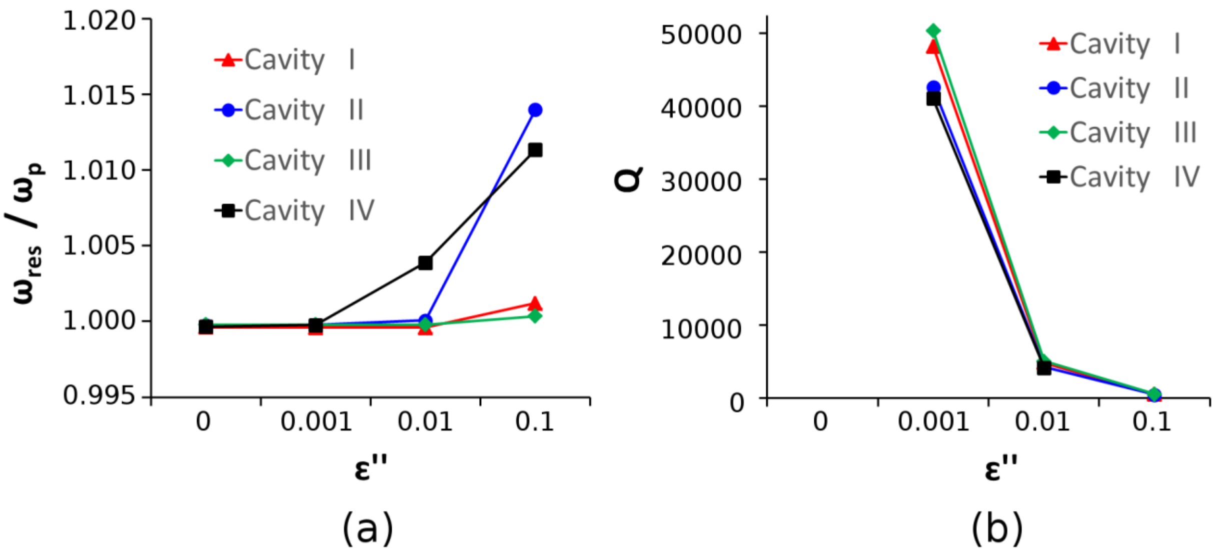

Supplementary Figure 5: Simulation results for (a) resonance frequency, normalized to the SiC plasma frequency, and (b) quality factor of the cavities represented in Fig. 2C of the main text for different amounts of loss at the SiC plasma frequency. The figure illustrates how the eigenfrequencies of all cavities collapse to the plasma frequency as losses decrease, while the quality factor diverges.

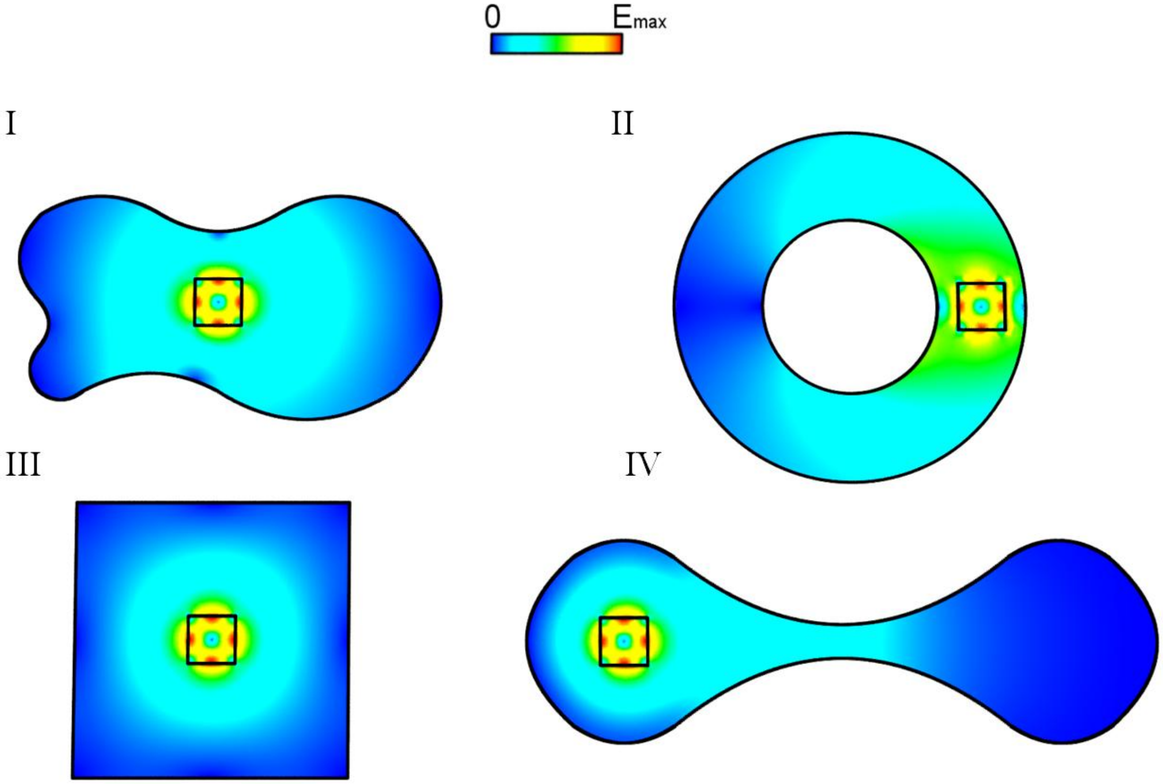

Supplementary Figure 6: Colormaps of the electric field magnitude distributions of resonant eigenmodes, obtained using numerical simulation, in four cavities consisting of a Si ($\varepsilon_i = 11.7$) square of side $l = 2.1525\ \mu$m, immersed in a SiC host of different shapes. The external boundaries of the cavities are shematically depicted in Figs. S1-S4, and they are identical to the ones employed in Fig. 2 of the main text. All cavities are bounded by perfectly electric conducting (PEC) walls. The eigenfrequencies and quality factors of these eigenmodes are shown in Supplementary Figure 7.

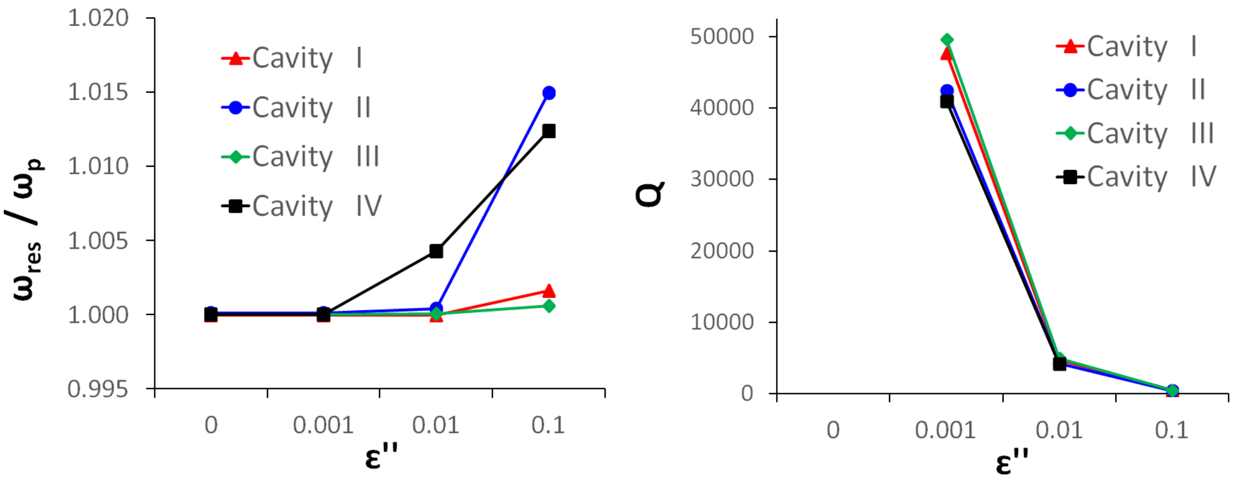

Supplementary Figure 7: Simulation results for (a) resonance frequency, normalized to the SiC plasma frequency, and (b) quality factor of the cavities represented in Supplementary Figure 6 for different amounts of loss at the SiC plasma frequency. The figure illustrates how the eigenfrequencies of all cavities collapse to the plasma frequency as losses decrease, while the quality factor diverges.

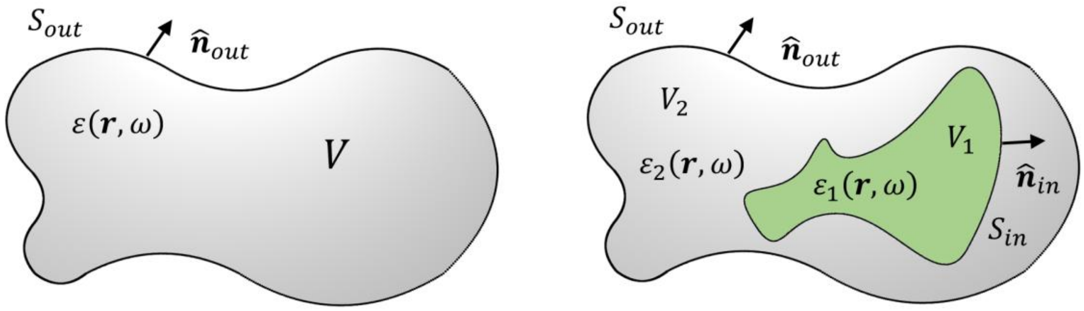

(a) **(b)**

Supplementary Figure 8: (a) Sketch of a generic 3D cavity of volume V bounded by the surface S_{out} with normal vector $\hat{\boldsymbol{n}}_{out}$, filled with a dielectric material with relative permittivity $\varepsilon(\boldsymbol{r},\omega)$. (a) Sketch of a generic 3D cavity bounded by the surface S_{out} with normal vector $\hat{\boldsymbol{n}}_{out}$, whose volume is composed by the union of two dielectric regions $V = V_1 + V_2$ with relative permittivity $\varepsilon_1(\boldsymbol{r},\omega)$ and $\varepsilon_2(\boldsymbol{r},\omega)$, respectively. Region V_1 is assumed to be a simply connected volume completely immersed within V_2 with surface S_{in} and normal vector $\hat{\boldsymbol{n}}_{in}$.

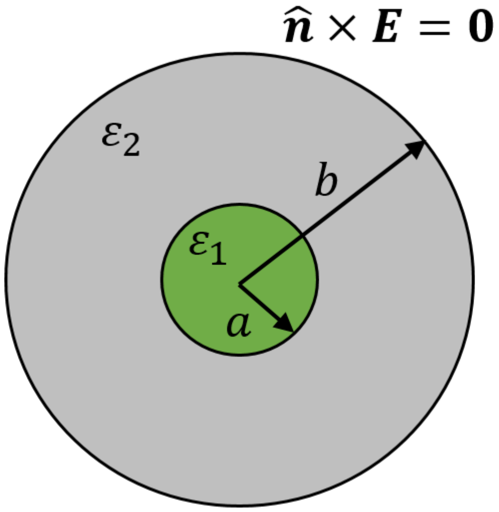

Supplementary Figure 9: Sketch of a cavity consisting of two concentric spheres, with internal and external radii equal to a and b, respectively, and with internal and external permittivities equal to ε_1 andε_2, respectively.

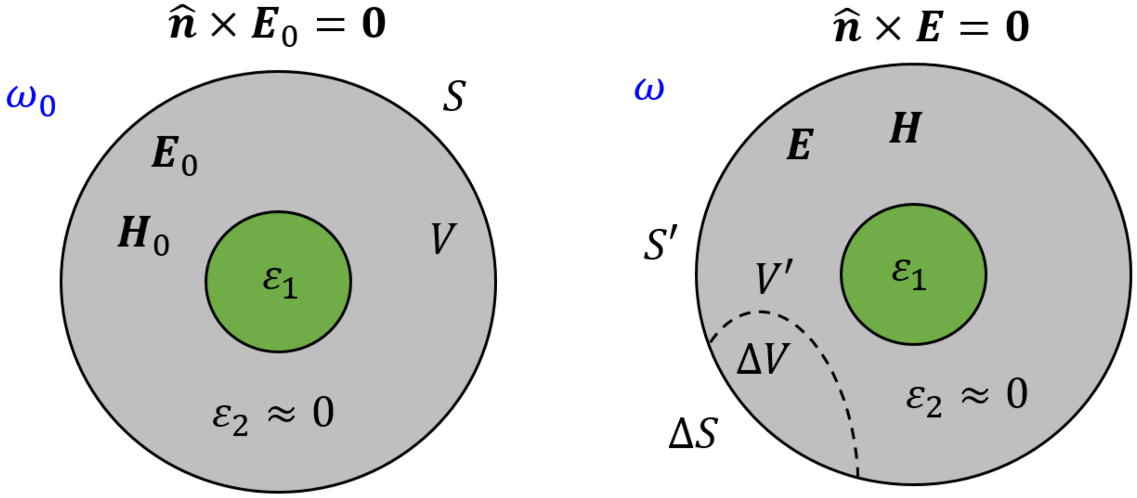

Supplementary Figure 10: (Left) Cavity characterized by volume V and surface S, supporting and eigenmode with fields $\boldsymbol{E}_0, \boldsymbol{H}_0$ at the eigenfrequency ω_0. (Right) Perturbed cavity with volume $V^{'} = V - \Delta V$ and volume $S^{'} = S - \Delta S$ supporting and eigenmode with fields $\boldsymbol{E}, \boldsymbol{H}$ at the eigenfrequency ω.

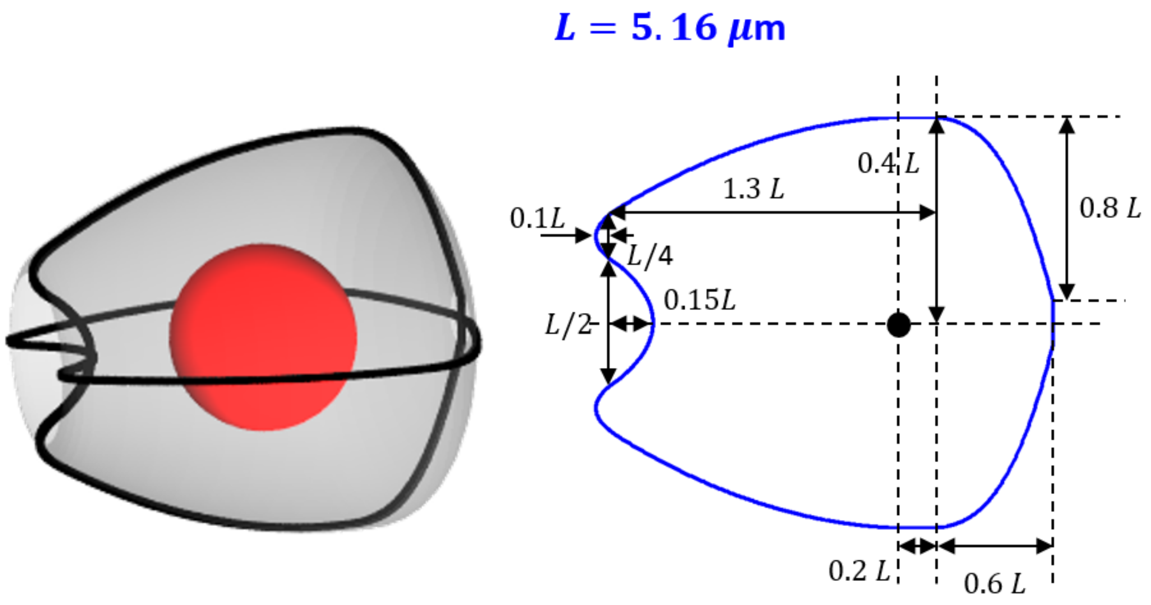

Supplementary Figure 11: Sketch (left) and dimensions (right) of the cavity studied in Fig. 3A-I of the main text. The cavity consists of a Si sphere ($\varepsilon_i = 11.7$) (shown in red) of radius $r_i = 2.155$ µm immersed in a SiC host (shown as grey background). The location of the sphere in the main cavity is indicated by a black dot.

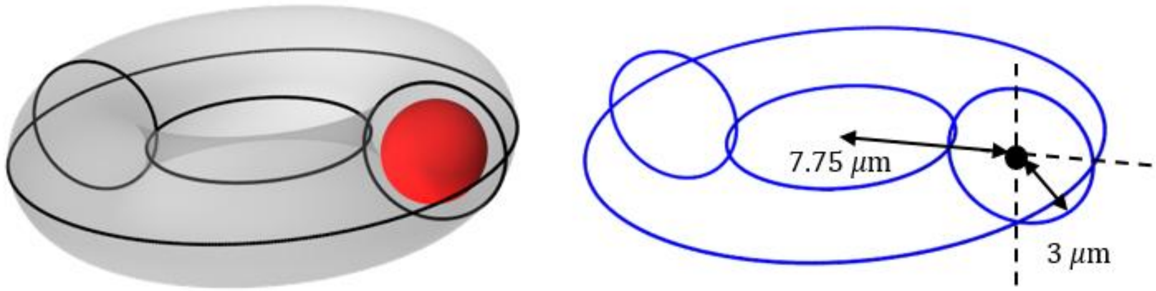

Supplementary Figure 12: Sketch (left) and dimensions (right) of the cavity studied in Fig. 3A-II of the main text. The cavity consists of a Si sphere ($\varepsilon_i = 11.7$) (shown in red) of radius $r_i = 2.155$ μm immersed in a SiC host (shown as grey background). The location of the sphere in the main cavity is indicated by a black dot.

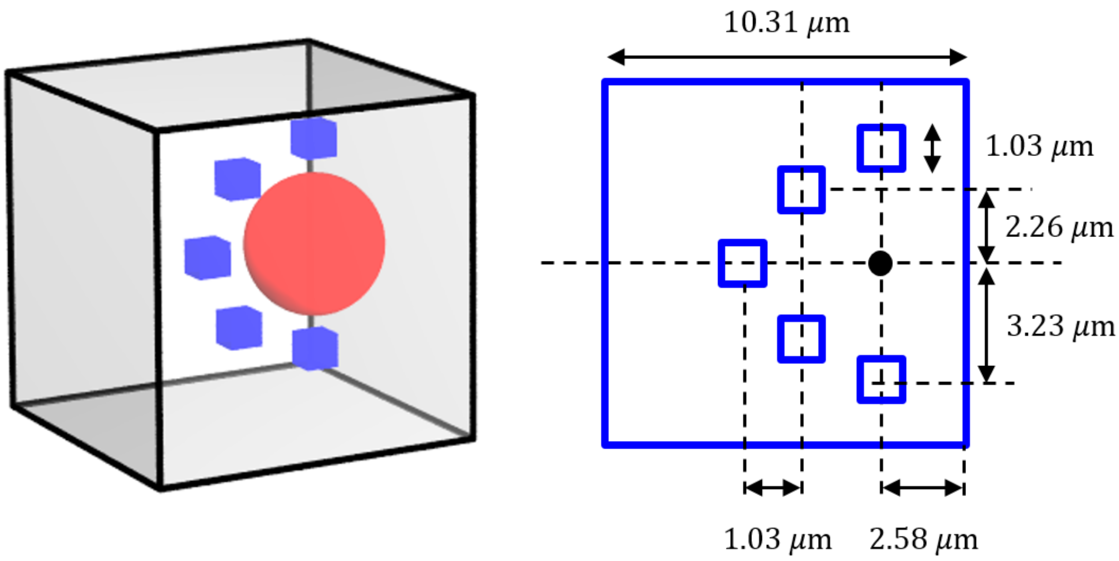

Supplementary Figure 13: Sketch (left) and dimensions (right) of the cavity studied in Fig. 3A-III of the main text. The cavity consists of a Si sphere ($\varepsilon_i = 11.7$) (shown in red) of radius $r_i = 2.155$ μm immersed in a SiC host (shown as grey background). The cavity also contains several additional cubic dielectric particles (shown in blue) with permittivity $\varepsilon_p = 2$. The location of the sphere in the main cavity is indicated by a black dot.

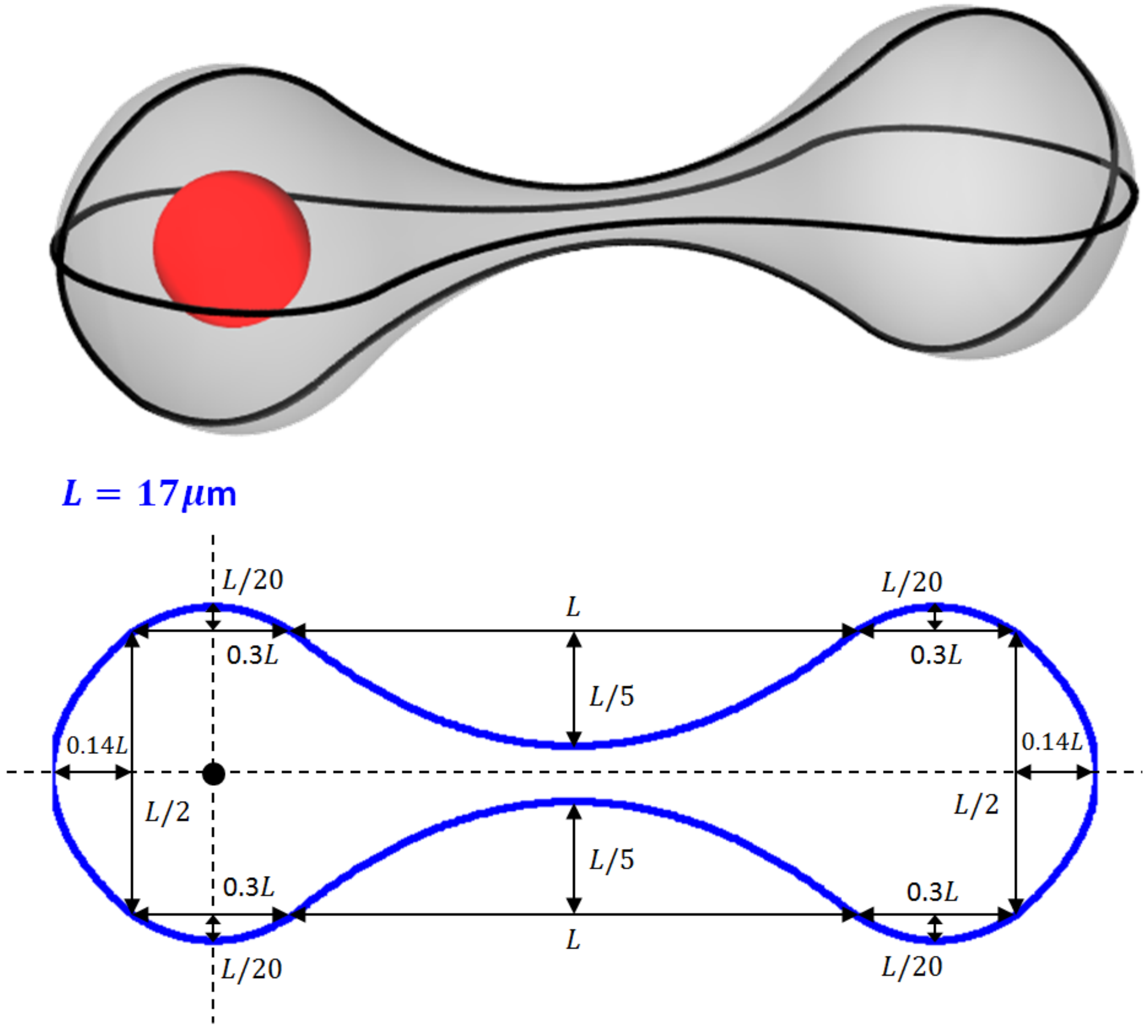

Supplementary Figure 14: Sketch (left) and dimensions (right) of the cavity studied in Fig. 3A-IV of the main text. The cavity consists of a Si sphere ($\varepsilon_i = 11.7$) (shown in red) of radius $r_i = 2.155$ µm immersed in a SiC host (shown as grey background). The location of the sphere in the main cavity is indicated by a black dot.

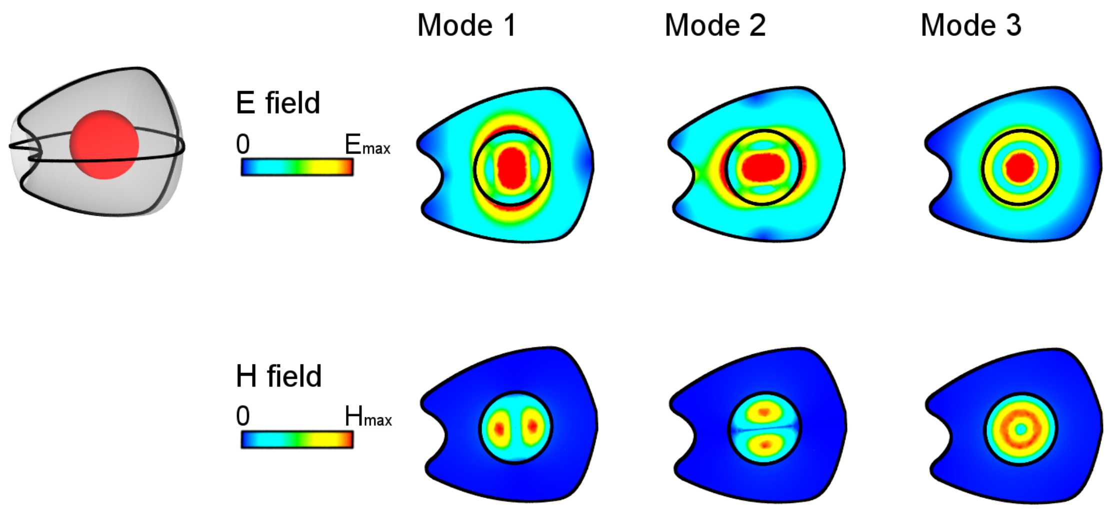

Supplementary Figure 15: Colormaps of the electric and magnetic field magnitude distributions of the degenerate modes excited in the cavity depicted in Fig. 3A-I of the main text. The eigenfrequencies and quality factors of these eigenmodes are shown in Fig. 3B, cavity I, of the main text.

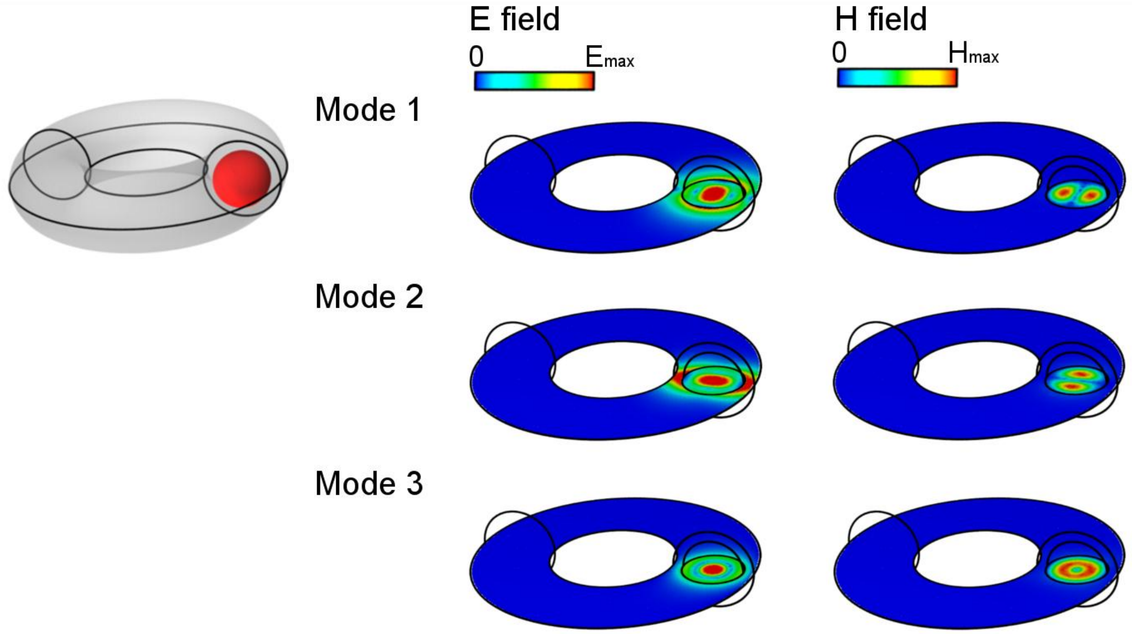

Supplementary Figure 16: Colormaps of the electric and magnetic field magnitude distributions of the degenerate modes excited in the cavity depicted in Fig. 3A-II of the main text. The eigenfrequencies and quality factors of these eigenmodes are shown in Fig. 3B, cavity II, of the main text.

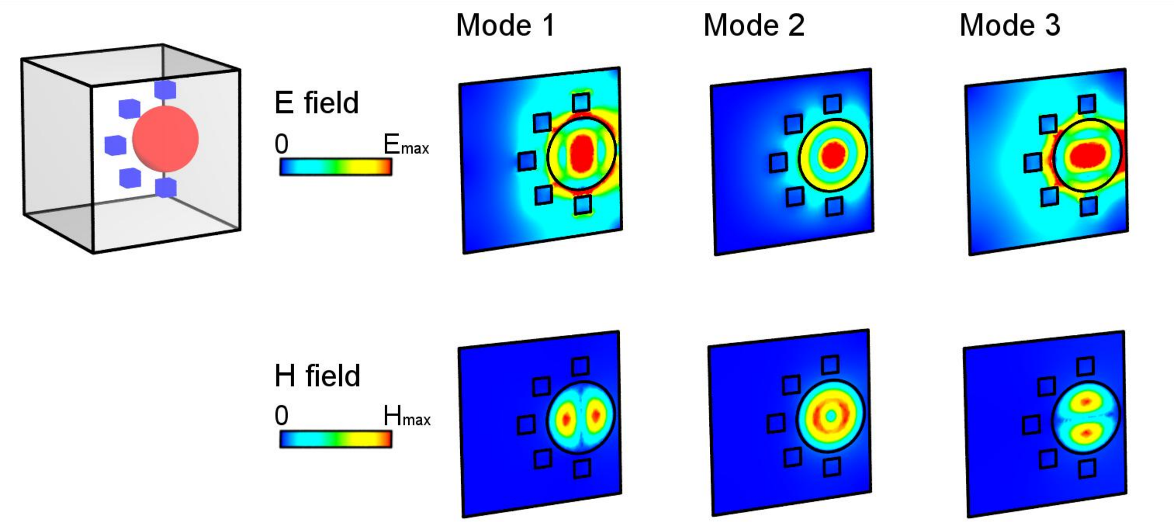

Supplementary Figure 17: Colormaps of the electric and magnetic field magnitude distributions of the degenerate modes excited in the cavity depicted in Fig. 3A-III, of the main text. The eigenfrequencies and quality factors of these eigenmodes are shown in Fig. 3B, cavity III, of the main text.

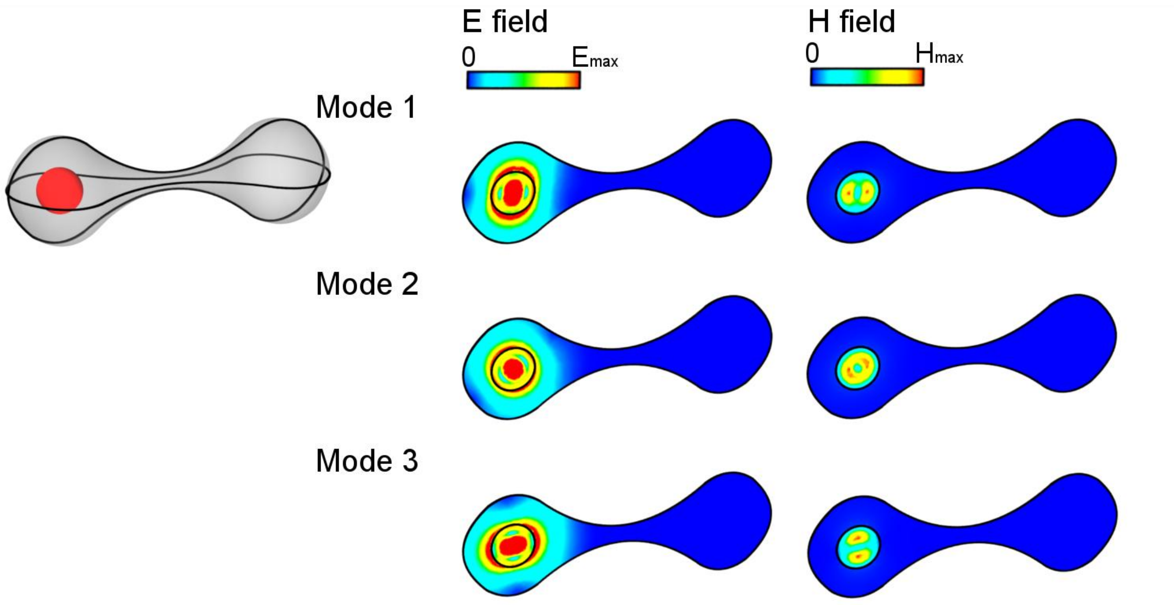

Supplementary Figure 18: Colormaps of the electric and magnetic field magnitude distributions of the degenerate modes excited in the cavity depicted in Fig. 3A-IV, of the main text. The eigenfrequencies and quality factors of these eigenmodes are shown in Fig. 3B, cavity IV, of the main text.

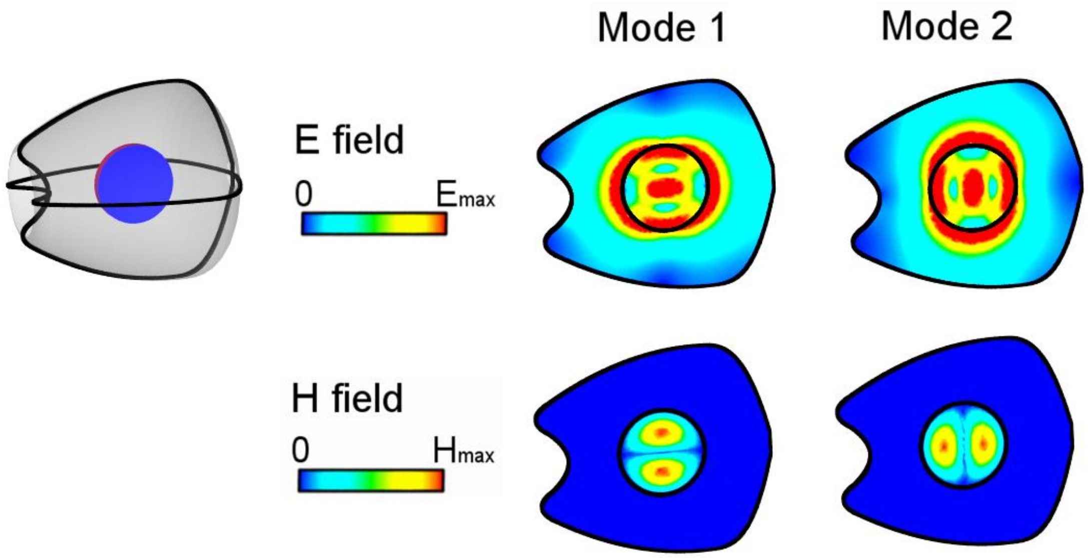

Supplementary Figure 19: Sketch of the cavity and colormaps of the electric and magnetic field magnitude distributions of the two degenerate modes excited close to the SiC plasma frequency. The cavity is identical to that studied in Fig. 3A-I of the main text, except that the internal spherical particle is replaced by a cylindrical particle (shown in red) of radius 1.828 μm and height 0.5 μm, whose top and bottom faces are covered with perfect-magnetic-conducting (PMC) layers (shown in blue). The radius of the cylinder has been fixed at the first zero of the Bessel function of the first kind and order 1, $J_1(k_i r_i) = 0$, so that it supports zero magnetic field on its lateral wall. The eigenfrequencies and quality factors of these eigenmodes are shown in Supplementary Figure 23, cavity I.

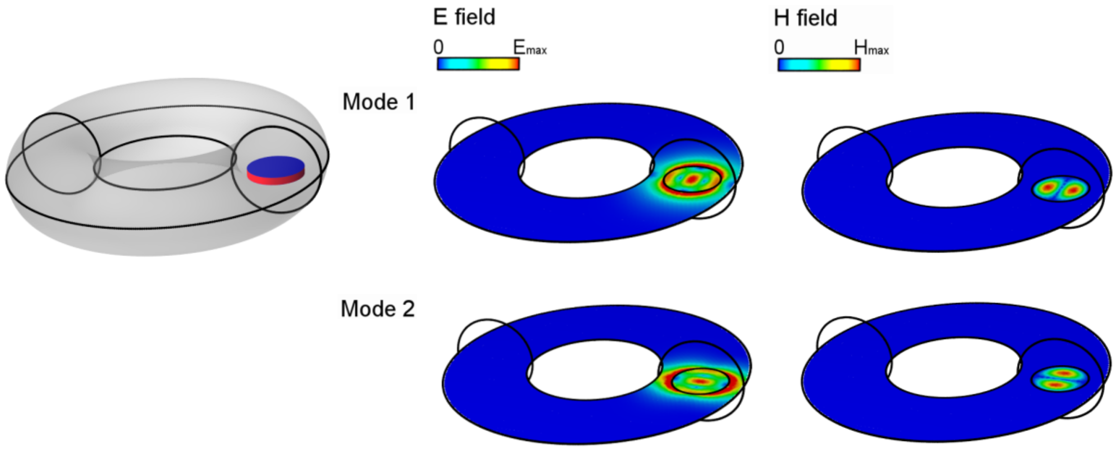

Supplementary Figure 20: Sketch of the cavity and colormaps of the electric and magnetic field magnitude distributions of the two degenerate modes excited close to the SiC plasma frequency. The cavity is identical to that studied in Fig. 3A-II of the main text, except that the internal spherical particle is replaced by a cylindrical particle (shown in red) of radius 1.828 μm and height 0.5 μm, whose top and bottom faces are covered with perfect-magnetic-conducting (PMC) layers (shown in blue). The radius of the cylinder has been fixed at the first zero of the Bessel function of the first kind and order 1, $J_1(k_i r_i) = 0$, so that it supports zero magnetic field on its lateral wall. The eigenfrequencies and quality factors of these eigenmodes are shown in Supplementary Figure 23, cavity II.

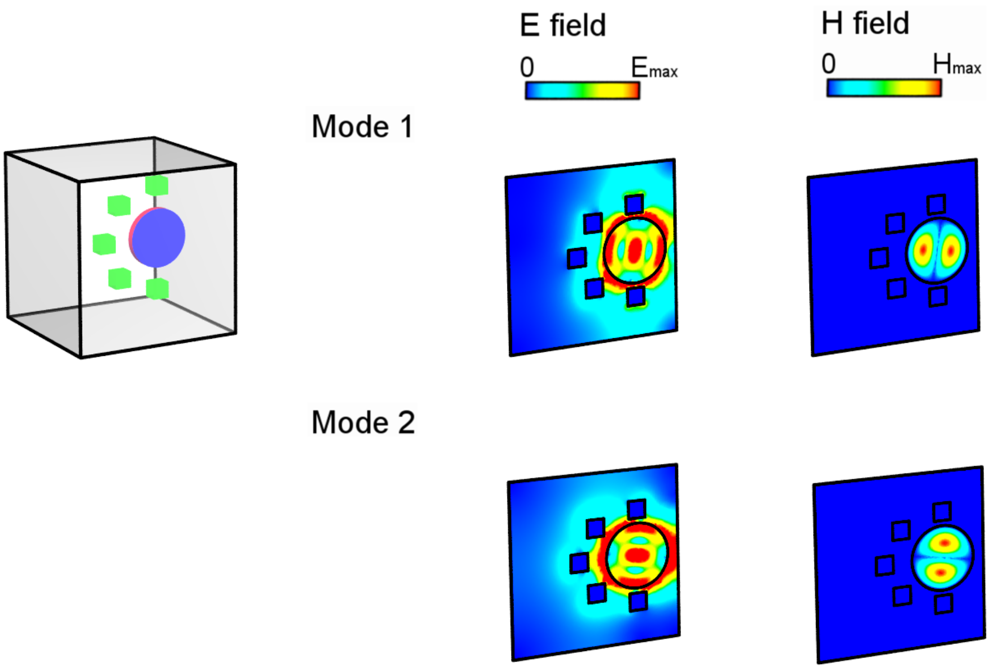

Supplementary Figure 21: Sketch of the cavity and colormaps of the electric and magnetic field magnitude distributions of the two degenerate modes excited close to the SiC plasma frequency. The cavity is identical to that studied in Fig. 3A-III of the main text, except that the internal spherical particle is replaced by a cylindrical particle (shown in red) of radius 1.828 μm and height 0.5 μm, whose top and bottom faces are covered with perfect-magnetic-conducting (PMC) layers (shown in blue). The radius of the cylinder has been fixed at the first zero of the Bessel function of the first kind and order 1, $J_1(k_i r_i) = 0$, so that it supports zero magnetic field on its lateral wall. The eigenfrequencies and quality factors of these eigenmodes are shown in Supplementary Figure 23, cavity III.

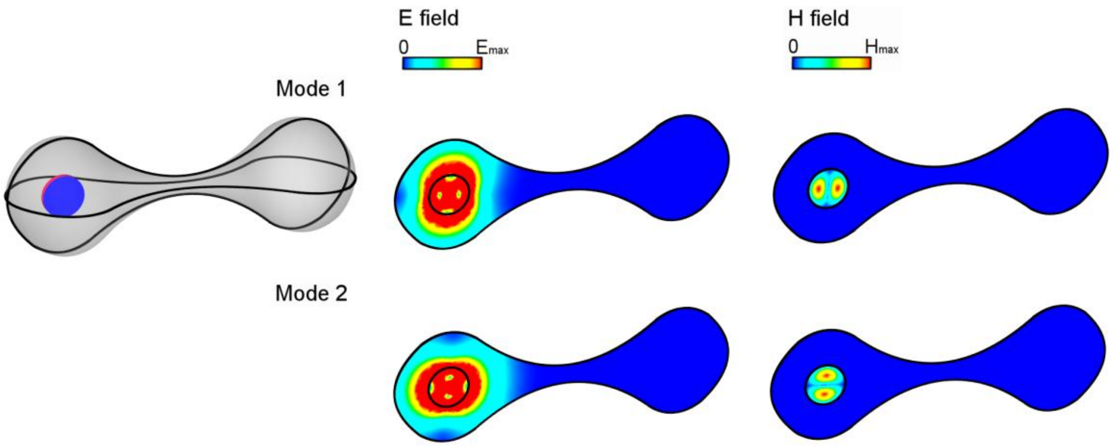

Supplementary Figure 22: Sketch of the cavity and colormaps of the electric and magnetic field magnitude distributions of the two degenerate modes excited close to the SiC plasma frequency. The cavity is identical to that studied in Fig. 3A-IV of the main text, except that the internal spherical particle is replaced by a cylindrical particle (shown in red) of radius 1.828 μm and height 0.5 μm, whose top and bottom faces are covered with perfect-magnetic-conducting (PMC) layers (shown in blue). The radius of the cylinder has been fixed at the first zero of the Bessel function of the first kind and order 1, $J_1(k_i r_i) = 0$, so that it supports zero magnetic field on its lateral wall. The eigenfrequencies and quality factors of these eigenmodes are shown in Supplementary Figure 23, cavity IV.

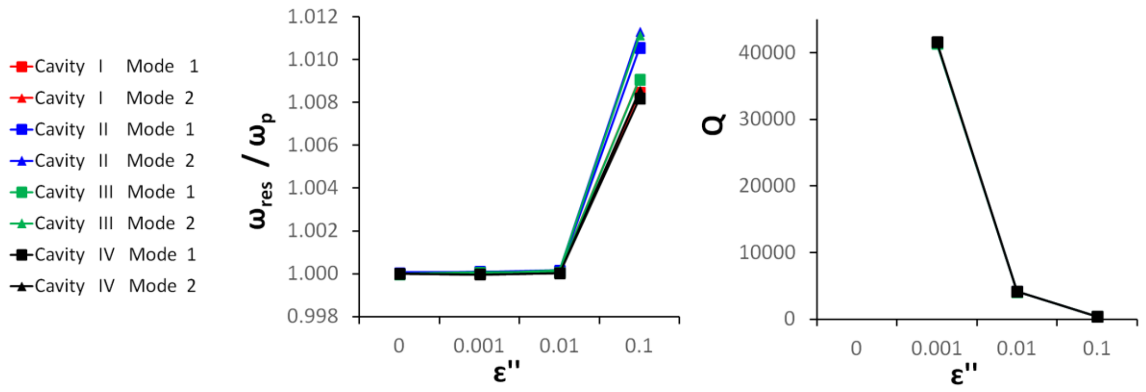

Supplementary Figure 23: Simulation results for (a) resonance frequency, normalized to the SiC plasma frequency, and (b) quality factor of the cavities represented in Supplementary Figures 19-22 for different amounts of loss at the SiC plasma frequency. The figure illustrates how the eigenfrequencies of all cavities collapse to the plasma frequency as losses decrease, while the quality factor diverges. In this case, the differences on the eigenfrequencies for finite losses are smaller than those reported in the cavities containg a spherical particle (Fig. 3 of the main text), whereas the differences on the quality factor are smaller. Both effects are associated to the strong fringing fields excited in the PMC top and bottom layers of the cylindrical paticle.

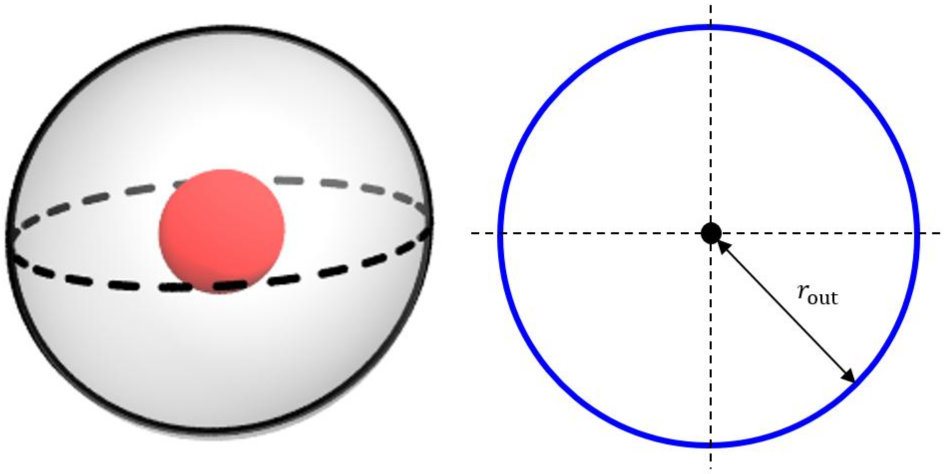

Supplementary Figure 24: Sketch (left) and dimensions (right) of the cavity studied in Figs. 4I to 4V of the main text. The cavity consists of a Si sphere ($\varepsilon_i = 11.7$) (shown in red) of radius $r_i = 1.507$ µm immersed in a SiC spherical host (shown as grey background) of different radii: 4I - $r_{\text{out}} = 3$ µm, 4II - $r_{\text{out}} = 4$ µm, III - $r_{\text{out}} = 5$ µm, IV - $r_{\text{out}} = 7.5$ µm and V - $r_{\text{out}} = 10$ µm. The location of the sphere in the main cavity is indicated by a black dot.

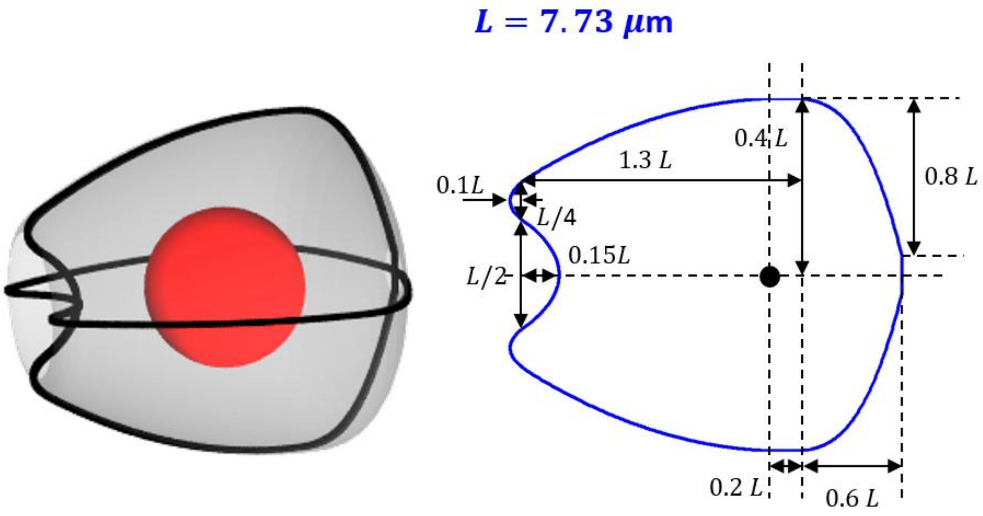

Supplementary Figure 25: Sketch (left) and dimensions (right) of the cavity studied in Fig. 4-VI of the main text. The cavity consists of a Si sphere ($\varepsilon_i = 11.7$) (shown as a red sphere) of radius $r_i = 1.507$ µm immersed in a SiC host (shown as grey background). The location of the sphere in the main cavity is indicated by a black dot.

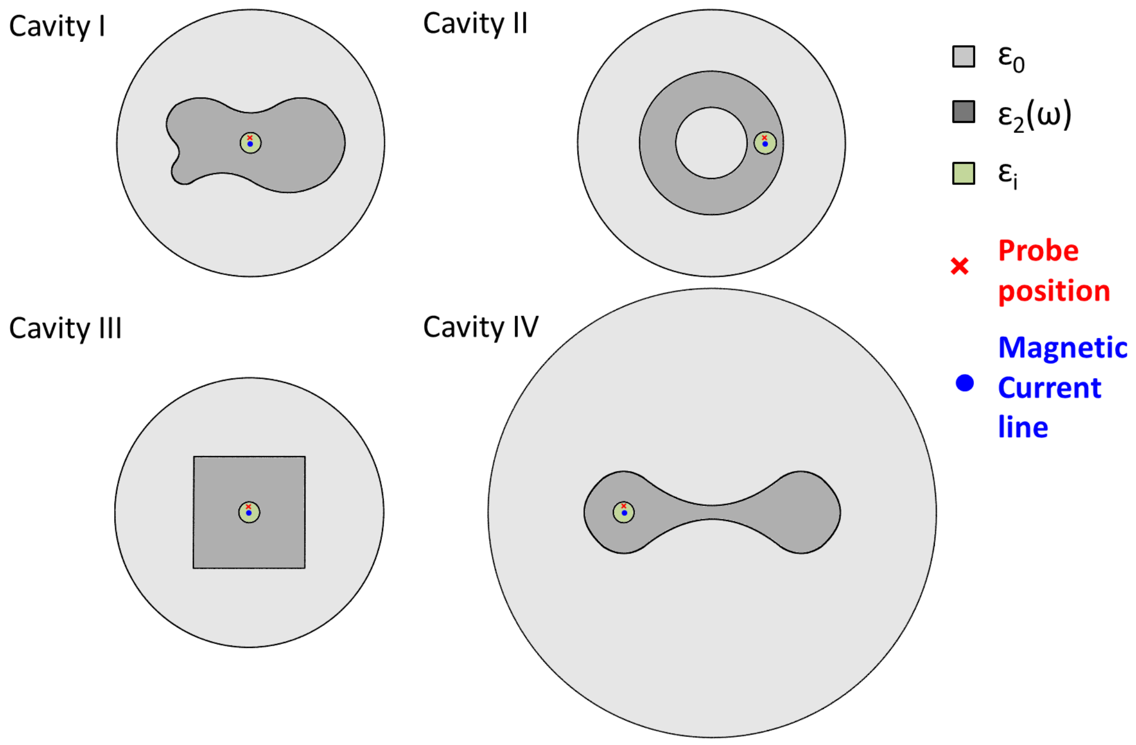

Supplementary Figure 26: Sketch of the simulation setup. The simulation setup consists of the 4 different 2D cavities studied in Fig. 2 of the main text (shown in dark grey), but with no PEC wall, and immersed within a vacuum circle (shown in light grey) terminated into a scattering boundary condition. (Please note that the dark solid line at the outer boundary of the vacuum circle is NOT the PEC wall, but it is a scattering boundary used in our simulator to imitate unbounded vacuum space scenario). The 2D cavities are excited with a 2D magnetic current line of 1 V (shown as a blue dot) perpendicular to the 2D plane, and positioned at the center of the dielectric cylinder (shown in green). The field intensity is monitored at the position indicated by the red cross, which is at 0.9 μm of the magnetic current source.

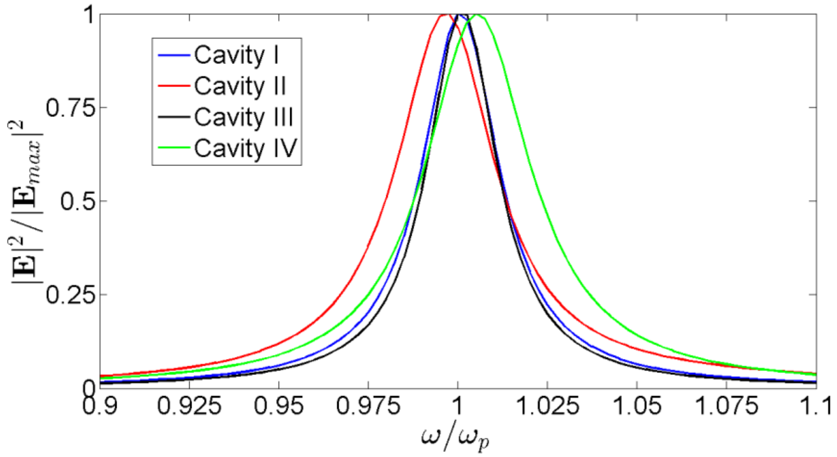

Supplementary Figure 27: Electric field intensity spectra at the probe position, normalized to its maximal value, for the 4 different 2D cavities studied in Fig. 2 of the main text, but with no PEC wall, and immersed in an unbounded vacuum space. The simulation setup is reported in Supplementary Figure 26.

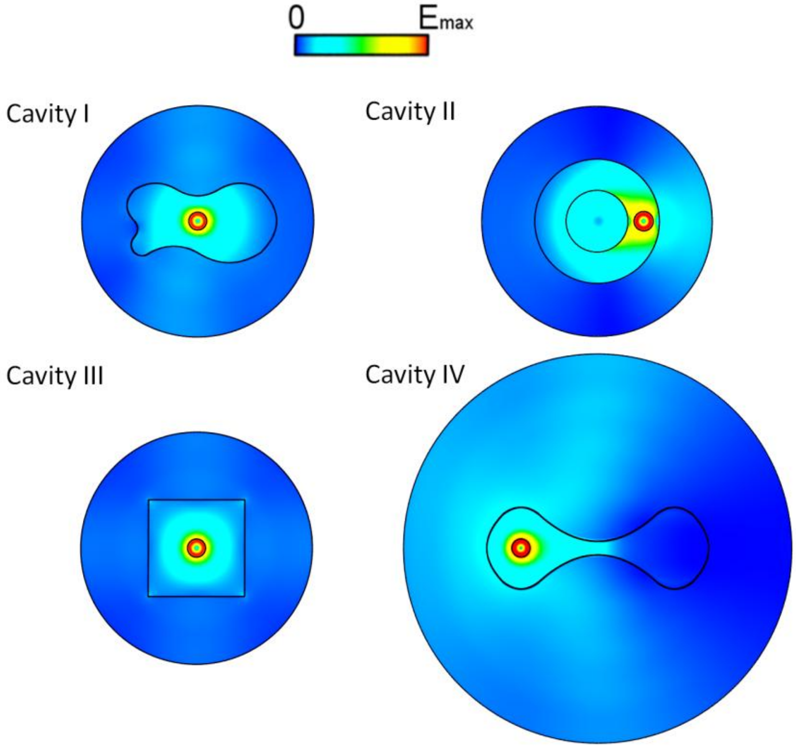

Supplementary Figure 28: Electric field distribution at the plasma frequency for the 4 different 2D cavities studied in Fig. 2 of the main text, but with no PEC wall, immersed in an unbounded vacuum space, and excited with a 2D magnetic current moment positioned at the center of the dielectric cylinder. The simulation setup is reported in Supplementary Figure 26.

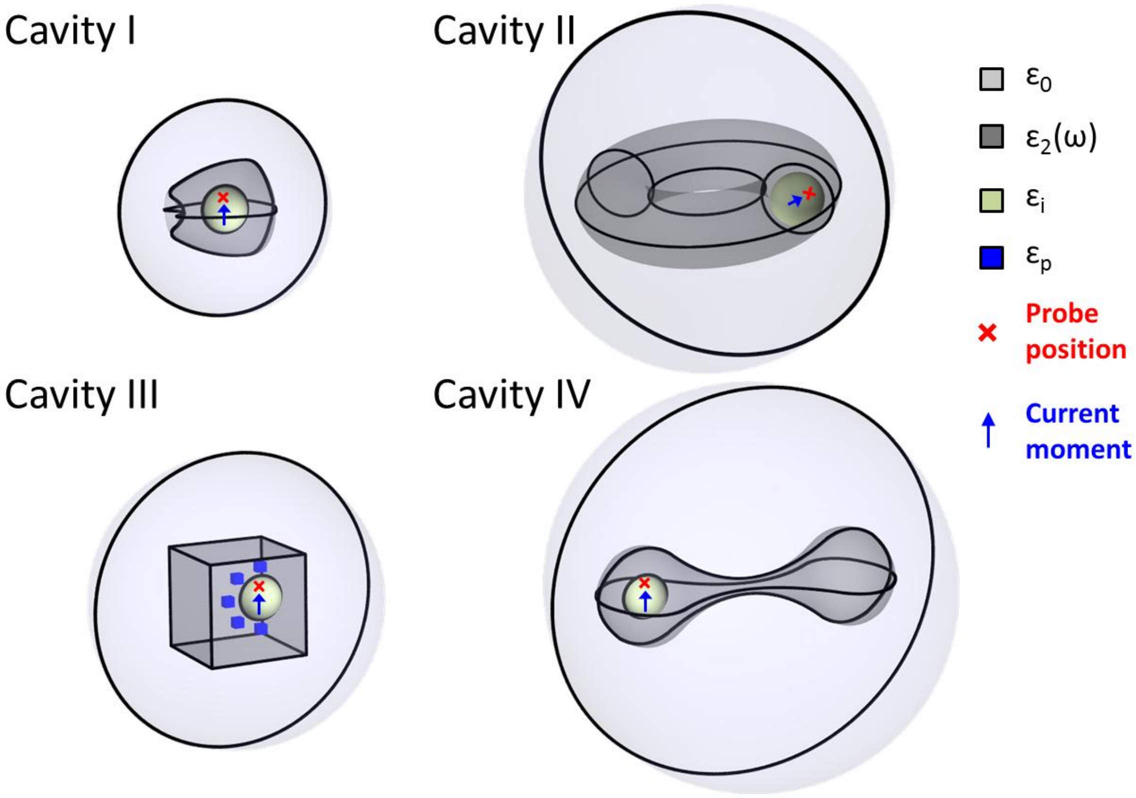

Supplementary Figure 29: Sketch of the simulation setup. The simulation setup consists of the 4 different 3D cavities studied in Fig. 3 of the main text (shown in dark grey), but with no PEC wall, and immersed within a vacuum sphere (shown in light grey) terminated into a scattering boundary condition. (Please note that the dark solid line at the outer boundary of the vacuum sphere is NOT the PEC wall, but it is a scattering boundary used in our simulator to imitate unbounded vacuum space scenario). The cavities are excited with a current moment of 1 A·m (shown as a blue arrow) positioned at the center of the dielectric sphere (shown in green). The field intensity is monitored at the position indicated by the red cross, which is at 0.5 μm on top of the current moment.

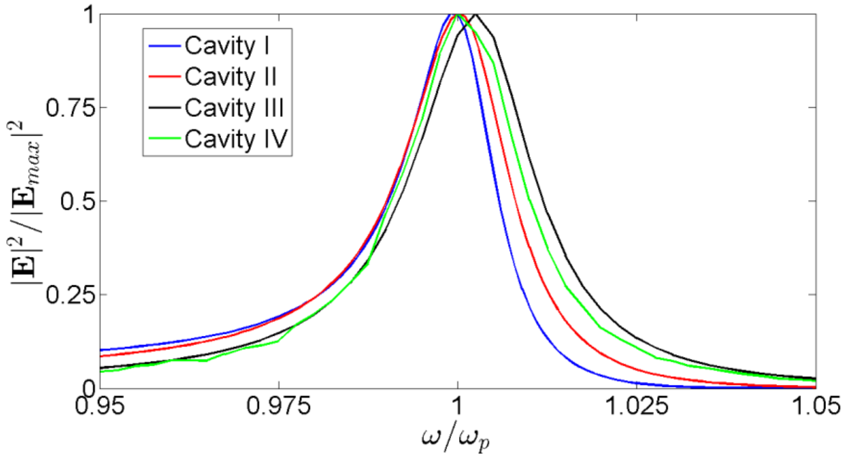

Supplementary Figure 30: Electric field intensity spectra at the probe position, normalized to its maximal value, for the 4 different 3D cavities studied in Fig. 3 of the main text, but with no PEC wall, and immersed in an unbounded vacuum space. The simulation setup is reported in Supplementary Figure 29.

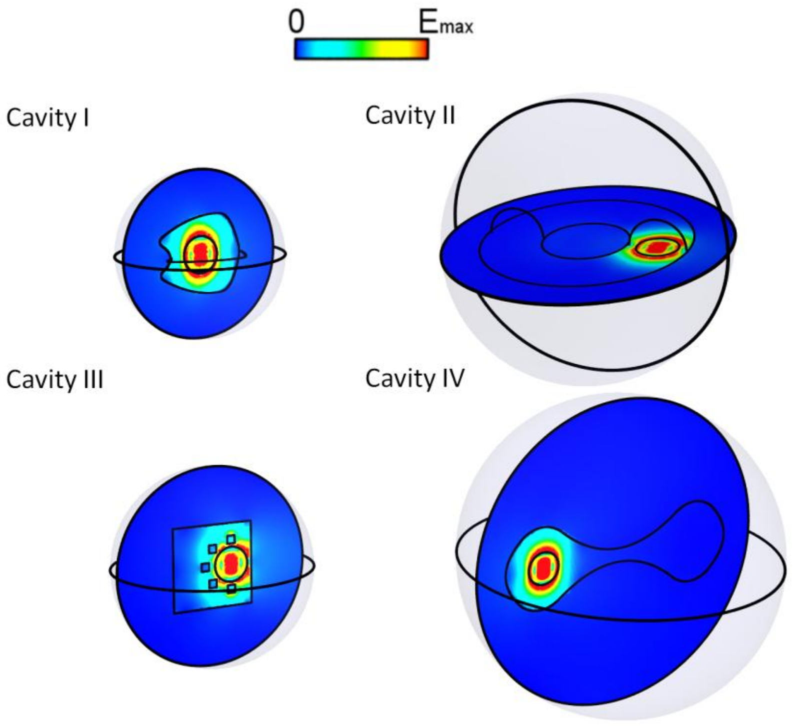

Supplementary Figure 31: Electric field magnitude distribution at the plasma frequency for the 4 different 3D cavities studied in Fig. 3 of the main text, but with no PEC wall, immersed in an unbounded vacuum space, and excited with a current moment positioned at the center of the dielectric sphere. The simulation setup is reported in Supplementary Figure 29.

Supplementary Note 1: Eigenfrequencies invariant under equi-areal transformations

Here we demonstrate the existence of 2D cavities supporting an eigenmode whose eigenfrequency is invariant under equi-areal transformations. These eigemodes are studied in Fig. 2 of the main text. To begin with, let us consider a 2D cavity of area $A = A_h + A_i$, composed of the union of a 2D dielectric particle and a 2D epsilon-near-zero (ENZ) host. The particle has cross-sectional area A_i and perimeter L_i, and it is characterized by relative permittivity ε_i. We assume that the particle is immersed in an ENZ host ($\varepsilon_h \simeq 0$) of cross-sectional area A_h externally bounded by perfectly electric conducting (PEC) walls (see Fig. 2A). Applying Faraday's law on the boundaries of the ENZ host only we get

$$\oint_{\partial A_h} \mathbf{E}\cdot d\mathbf{l} = \oint_{\partial A} \mathbf{E}\cdot d\mathbf{l} - \oint_{\partial A_i} \mathbf{E}\cdot d\mathbf{l} = i\omega\mu_0 \int_{A_h} \mathbf{H}\cdot\widehat{\mathbf{z}}\, dS \tag{1}$$

The boundary condition $\widehat{\mathbf{n}}\times\mathbf{E} = \mathbf{0}$ imposes that the circulation of the electric field on ∂A is zero. Following and analysis similar to that in Ref. [5], we start by noting that the magnetic field within the ENZ host must be constant, $\mathbf{H} = \widehat{\mathbf{z}}\, H_z^h$, in order to ensure a finite electric field $\mathbf{E} = i\left(\omega\varepsilon_0\varepsilon_h\right)^{-1}\nabla\times\mathbf{H} = i\left(\omega\varepsilon_0\varepsilon_h\right)^{-1}\nabla H_z^h \times\widehat{\mathbf{z}}$. Therefore, the characteristic equation that must be satisfied in order to support an eigenmode at the plasma frequency can be written as follows:

$$\omega = \frac{i}{\mu_0}\frac{L_i}{A_h} Z_S \tag{2}$$

where

$$Z_S = \frac{\oint_{\partial A_i}\mathbf{E}\cdot d\mathbf{l}}{H_z^h\, L_i} \tag{3}$$

Z_S represents the surface impedance of the internal particle, i.e., the line integral of the electric field divided by the surface current. It can be readily checked that the surface impedance (3) is completely determined by the geometrical and material properties of the particle, i.e., Z_S is independent on the geometry of the cavity in which it is immersed, as long as it is fully immersed in an ENZ host. To prove this point, we recall that, in this 2D problem with a $\widehat{\mathbf{z}}$-linearly polarized magnetic field, the electric field is the determined by the gradient of the magnetic field, $\mathbf{E} = i\left(\omega\varepsilon_0\varepsilon_i\right)^{-1}\nabla H_z(\boldsymbol{\rho})\times\widehat{\mathbf{z}}$. Therefore, the electric field within the particle, and hence the line integral in (3) are determined by the magnetic field distribution within the particle $H_z(\boldsymbol{\rho})$. Moreover, since the magnetic field is linearly polarized, its spatial distribution is given by the solution to the scalar Helmholtz equation

$$\nabla^2 H_z(\boldsymbol{\rho}) + \frac{\omega^2}{c^2}\varepsilon_i\, H_z(\boldsymbol{\rho}) = 0 \tag{4}$$

The solution to this wave equation must also satisfy the boundary condition imposed by the continuity of the fields on the boundary of the particle:

$$H_z(\boldsymbol{\rho}) = H_z^h \;\text{ on }\; \partial A_i \tag{5}$$

In other words, the magnetic field within the rod is given by the solution to the internal Dirichlet problem for the Helmholtz equation (4) with boundary condition (5). In general, the distribution of the magnetic field on the surface of the particle depends on the properties of the space external to the particle. In this manner, the boundary condition (5) is in general a continuous function that depends on the properties of the space external to the cavity. However, when the 2D particle is immersed in a 2D ENZ medium, the magnetic field at the boundary of the particle is contstant, $H_z(\boldsymbol{\rho}) = H_z^h$, independently of any other characteristic of the space external to the particle. In other words, the magnetic field within the rod is given by the solution to the internal Dirichlet problem of the Helmholtz equation, applied to the case in which the function at the boundary is a constant. Consequently, the spatial distributions of the fields within the particle are independent of the external shape of the ENZ host.

As for their magnitude, since the equations involved are linear, the field inside the particle will be proportional to the field outside. Therefore, it is evident from (3) that the surface impedance is completely determined by the geometrical and material properties of the particle, and it is independent of the cavity. Subsequently, it is clear from (2) that the existence of an eigenmode at the plasma frequency is completely determined by the properties of the particle, encapsulated in the surface impedance, Z_S, and the cross-sectional area of the ENZ host, A_h. Therefore, the existence of an eigenmode is independent of the shape of the external boundary of the cavity as long as its area is kept constant. This includes changes in the topology of the cavity such as including holes. The only requirement is that the internal particle and the overall area of the ENZ host are not affected by the geometrical transformation.

As a specific example, let us consider a a cylindrical particle of radius r_i. In this case, it is convenient to write the magnetic field within the cylinder as a series of cylindrical harmonics

$$H_z^i = \sum_{n=-\infty}^{\infty} i\omega\, C_n\, J_n\left(k_i \rho\right)\, e^{in\phi} \tag{6}$$

where the C_n elements are complex constants determining the magnitude of the magnetic field and $k_i = k_0\sqrt{\varepsilon_i}$ is the propagation constant within the cylinder. $J_n\left(x\right)$ is the cylindrical Bessel function of the first kind and order n. Recall that the magnetic field in the ENZ region is constant. Therefore, in order to satisfy the continuity of the magnetic field on the surface of the cylinder only the $n = 0$ mode, with no azimuthal variation, can be excited. Exceptions occur in those cases in which the magnetic field in the ENZ host is zero, which are considered in the next section. Thus, by imposing the continuity of the tangential fields for a non-zero magnetic field the line integral of the electric field on the surface of the cylinder can be written as follows:

$$\oint_{\partial A_i} \mathbf{E}\cdot d\mathbf{l} = -iH_z^h \eta_i \left(2\pi r_i\right) \frac{J_0'\left(k_i r_i\right)}{J_0\left(k_i r_i\right)} \tag{7}$$

where $\eta_i = \eta_0/\sqrt{\varepsilon_i}$ is the medium intrinsic impedance within the cylinder. Consequently, the surface impedance is given by

$$Z_S = -i\,\eta_i \frac{J_0'\left(k_i r_i\right)}{J_0\left(k_i r_i\right)} \tag{8}$$

Next, the characteristic equation that determines the existence of an eigenmode at the plasma frequency is found by introducing (8) into (2)

$$\omega = \frac{c}{\sqrt{\varepsilon_i}} \frac{2\pi r_i}{A_h} \frac{J_0'\left(k_i r_i\right)}{J_0\left(k_i r_i\right)} \tag{9}$$

The theory above has been numerically validated by using a commercial electromagnetic solver (see Methods). Specifically, we have included numerical examples of several 2D ENZ cavities containing a dielectric cylinder (Fig. 2 of the main text, and Supplementary Figures 1-5), as well as several 2D cavities in which the internal particle consists of a dielectric cylinder with square cross-section (Supplementary Figures 6-7).

Supplementary Note 2: Spatially "electrostatic" fields in epsilon-near-zero media:

Here we demonstrate that epsilon-near-zero ENZ media support $\exp(-i\omega t)$ time-varying spatially electrostatic field distributions (i.e., $\mathbf{H} = \mathbf{0} \rightarrow \nabla \times \mathbf{E} = \mathbf{0}$). To begin with, we can inspect time-harmonic Maxwell curl equations in a host medium of relative permittivity ε_h:

$$\nabla \times \mathbf{H} = -i\omega\varepsilon_0\varepsilon_h\mathbf{E} \tag{10}$$

$$\nabla \times \mathbf{E} = i\omega\mu_0\mathbf{H} \tag{11}$$

It is clear from (10) that, when $\varepsilon_h = 0$, the medium can support non-zero time-varying electric fields even when the magnetic field is zero $\mathbf{H} = \mathbf{0}$. Naturally, it follows from (11) that the electric field must be irrotational $\nabla \times \mathbf{E} = \mathbf{0}$.

The existence of $\exp(-i\omega t)$ time-varying spatially electrostatic modes can also be derived as a limiting case of the usual solution of time-harmonic Maxwell curl equations. Without loss of generality, the electric and magnetic fields can be written as the sum of transversal magnetic (TM) and transversal electric (TE) fields, i.e., $\mathbf{E} = \mathbf{E}^{TM} + \mathbf{E}^{TE}$ and $\mathbf{H} = \mathbf{H}^{TM} + \mathbf{H}^{TE}$. In addition, each term can be written by using a multipolar decomposition in terms of Tesseral harmonics: [6]

$$\mathbf{E}^{TM} = -i\sum_{\{q\}}\left\{n\left(n+1\right)\frac{A_{nml}^{TM}\widehat{J}_n\left(kr\right) + B_{nml}^{TM}\widehat{Y}_n\left(kr\right)}{\left(kr\right)^2}\mathbf{T}_{nml}\left(\widehat{\mathbf{r}}\right) + \frac{A_{nml}^{TM}\widehat{J}'_n\left(kr\right) + B_{nml}^{TM}\widehat{Y}'_n\left(kr\right)}{kr}\boldsymbol{\psi}_{nml}\left(\widehat{\mathbf{r}}\right)\right\} \tag{12}$$

$$\mathbf{H}^{TM} = \frac{1}{\eta}\sum_{\{q\}}\frac{A_{nml}^{TM}\widehat{J}_n\left(kr\right) + B_{nml}^{TM}\widehat{Y}_n\left(kr\right)}{kr}\boldsymbol{\varphi}_{nml}\left(\widehat{\mathbf{r}}\right) \tag{13}$$

$$\mathbf{E}^{TE} = \sum_{\{q\}}\frac{A_{nml}^{TE}\widehat{J}_n\left(kr\right) + B_{nml}^{TE}\widehat{Y}_n\left(kr\right)}{kr}\boldsymbol{\varphi}_{nml}\left(\widehat{\mathbf{r}}\right) \tag{14}$$

$$\mathbf{H}^{TE} = \frac{i}{\eta}\sum_{\{q\}}\left\{n\left(n+1\right)\frac{A_{nml}^{TE}\widehat{J}_n\left(kr\right) + B_{nml}^{TE}\widehat{Y}_n\left(kr\right)}{\left(kr\right)^2}\mathbf{T}_{nml}\left(\widehat{\mathbf{r}}\right) + \frac{A_{nml}^{TE}\widehat{J}'_n\left(kr\right) + B_{nml}^{TE}\widehat{Y}'_n\left(kr\right)}{kr}\boldsymbol{\varphi}_{nml}\left(\widehat{\mathbf{r}}\right)\right\} \tag{15}$$

where $\{q\} = \{n, m, l\}$ is a multi-index defined so that the sum runs over all spherical multipoles:

$$\sum_{\{q\}} = \sum_{n=1}^{\infty}\sum_{m=0}^{n}\sum_{l=e,o} \tag{16}$$

In such a decomposition, the radial dependency of the field is described via the Schelkunoff form of the spherical Bessel functions, $\widehat{J}_n\left(kr\right)$ and $\widehat{Y}_n\left(kr\right)$, where, for example, $\widehat{J}_n\left(x\right) = \sqrt{\pi x/2}\,J_{n+1/2}\left(x\right)$ with $J_n\left(x\right)$ being the Bessel function of the first kind and order n [6]. On the other hand, the angular dependency is described by Tesseral harmonics $\mathbf{T}_{nml}\left(\widehat{\mathbf{r}}\right)$ and linear combinations of its derivatives, $\boldsymbol{\psi}_{nml}\left(\widehat{\mathbf{r}}\right)$ and $\boldsymbol{\varphi}_{nml}\left(\widehat{\mathbf{r}}\right)$, given by:

$$\mathbf{T}_{nm\binom{e}{o}}\left(\widehat{\mathbf{r}}\right) = \widehat{\mathbf{r}}\,P_n^m\left(\cos\theta\right)\begin{pmatrix}\cos m\phi\\ \sin m\phi\end{pmatrix} \tag{17}$$

$$\boldsymbol{\psi}_{nml}\left(\widehat{\mathbf{r}}\right) = \widehat{\boldsymbol{\theta}}\,\partial_\theta T_{nml}\left(\widehat{\mathbf{r}}\right) + \widehat{\boldsymbol{\phi}}\frac{\partial_\phi T_{nml}\left(\widehat{\mathbf{r}}\right)}{\sin\theta} \tag{18}$$

$$\boldsymbol{\varphi}_{nml}\left(\widehat{\mathbf{r}}\right) = \boldsymbol{\psi}_{nml}\left(\widehat{\mathbf{r}}\right) \times \widehat{\mathbf{r}} \tag{19}$$

When the host medium is ENZ ($\varepsilon \to 0$, so $\eta \to \infty$ and $k \to 0$), the TM fields (20)-(21) reduce to

$$\mathbf{E}^{TM} = -i \sum_{\{q\}} \left\{ n\,(n+1) \left[\frac{1}{(2n+1)!!} \left(k^{n-1} A_{nml}^{TM}\right) r^{n-1} - (2n-1)!! \left(\frac{B_{nml}^{TM}}{k^{n+2}} \right) \frac{1}{r^{n+2}} \right] \mathbf{T}_{nml}\left(\widehat{\mathbf{r}}\right) \right.$$

$$\left. + \left[\frac{n+1}{(2n+1)!!} \left(k^{n-1} A_{nml}^{TM}\right) r^{n-1} + n\,(2n-1)!! \left(\frac{B_{nml}^{TM}}{k^{n+2}} \right) \frac{1}{r^{n+2}} \right] \boldsymbol{\psi}_{nml}\left(\widehat{\mathbf{r}}\right) \right\} \tag{20}$$

$$\mathbf{H}^{TM} = \frac{1}{\omega \mu_0} \sum_{\{q\}} \left[\frac{1}{(2n+1)!!} \left(k^{n+1} A_{nml}^{TM}\right) r^{n} - (2n-1)!! \left(\frac{B_{nml}^{TM}}{k^{n}} \right) \frac{1}{r^{n-1}} \right] \boldsymbol{\varphi}_{nml}\left(\widehat{\mathbf{r}}\right) \tag{21}$$

It is apparent from (20) that in order to $\mathbf{E}^{TM}$ to be finite but non-zero it is required $A_{nml}^{TM} \propto k^{-n+1}$ and $B_{nml}^{TM} \propto k^{n+2}$. However, it is also clear from (13) that this implies a zero magnetic field $\mathbf{H}^{TM} = \mathbf{0}$. In this manner, the multipolar decomposition ratifies the existence of spatially electrostatic fields in ENZ media. In addition, this decomposition gives some hints on how to excite these fields. In general, sources of TM multipoles and their linear combinations generate time-varying spatially electrostatic fields in ENZ media.

On the other hand, the TE fields (22)-(23) in ENZ media can be written as follows:

$$\mathbf{E}^{TE} = \sum_{\{q\}} \left[\frac{1}{(2n+1)!!} \left(k^{n} A_{nml}^{TE}\right) r^{n} - (2n-1)!! \left(\frac{B_{nml}^{TE}}{k^{n+1}} \right) \frac{1}{r^{n+1}} \right] \boldsymbol{\varphi}_{nml}\left(\widehat{\mathbf{r}}\right) \tag{22}$$

$$\mathbf{H}^{TE} = \frac{i}{\omega \mu_0} \sum_{\{q\}} \left\{ n\,(n+1) \left[\frac{1}{(2n+1)!!} \left(k^{n} A_{nml}^{TE}\right) r^{n-1} - (2n-1)!! \left(\frac{B_{nml}^{TE}}{k^{n+1}} \right) \frac{1}{r^{n+2}} \right] \mathbf{T}_{nml}\left(\widehat{\mathbf{r}}\right) \right.$$

$$\left. \left[\frac{n+1}{(2n+1)!!} \left(k^{n} A_{nml}^{TE}\right) r^{n-1} + n\,(2n-1)!! \left(\frac{B_{nml}^{TE}}{k^{n+1}} \right) \frac{1}{r^{n+2}} \right] \boldsymbol{\psi}_{nml}\left(\widehat{\mathbf{r}}\right) \right\} \tag{23}$$

In this case, in order to obtain a finite but non-zero elecric field $\mathbf{E}^{TE}$ requires $A_{nml}^{TE} \propto k^{-n}$ and $B_{nml}^{TM} \propto k^{n+1}$. However, by contrast with the TM modes, this does not implies a zero mangetic field $\mathbf{H}^{TE}$.

Supplementary Note 3: Spatially "electrostatic" eigenmodes

Here we demonstrate the existence of eigenmodes with spatially "electrostatic" field distributions, whose eigenfrequencies are independent with respect to geometrical deformations of the external boundaries of the cavity. These eigemodes are numerically studied in Fig. 3 of the main text. To this end, let us consider first a generic 3D cavity of volume V bounded by the surface S_{out} with outward normal unit vector $\widehat{\mathbf{n}}_{\mathrm{out}}$(see Supplementary Figure 8a). We define an eigenmode with eigenfrequency ω as a field distribution $\mathbf{E}(\mathbf{r},\omega)$ which is a solution to the time-harmonic wave equation

$$\nabla \times \nabla \times \mathbf{E}(\mathbf{r},\omega) - \frac{\omega^2}{c^2}\,\varepsilon(\mathbf{r},\omega)\;\mathbf{E}(\mathbf{r},\omega) = \mathbf{0} \tag{24}$$

subject to the boundary condition:

$$\widehat{\mathbf{n}}_{\mathrm{out}} \times \mathbf{E}(\mathbf{r},\omega) = \mathbf{0} \tag{25}$$

Next, let the cavity be constructed by the union of two regions, $V = V_1 + V_2$, with homogenous permittivities $\varepsilon(\mathbf{r},\omega) = \varepsilon_1(\omega)$ for $\mathbf{r} \in V_1$, and $\varepsilon_2(\mathbf{r},\omega) = \varepsilon_2(\omega)$ for $\mathbf{r} \in V_2$ (see Supplementary Figure 8b). In addition, we select V_1 as a simply connected volume (i.e., the particle) bounded by the surface S_{in} with outward normal unit vector $\widehat{\mathbf{n}}_{\mathrm{in}}$. We assume that the particle V_1 is completely immersed within V_2 (i.e., the background host). In this manner, the eigenmodes of the cavity at the eigenfrequency ω correspond to the electric field distributions $\mathbf{E}(\mathbf{r},\omega) = \mathbf{E}_1(\mathbf{r},\omega)$ for $\mathbf{r} \in V_1$ and $\mathbf{E}(\mathbf{r},\omega) = \mathbf{E}_2(\mathbf{r},\omega)$ for $\mathbf{r} \in V_2$ that satisfy the wave equations

$$\nabla \times \nabla \times \mathbf{E}_2(\mathbf{r},\omega) - \frac{\omega^2}{c^2}\,\varepsilon_2(\omega)\;\mathbf{E}_2(\mathbf{r},\omega) = \mathbf{0} \tag{26}$$

$$\nabla \times \nabla \times \mathbf{E}_1(\mathbf{r},\omega) - \frac{\omega^2}{c^2}\,\varepsilon_1(\omega)\;\mathbf{E}_1(\mathbf{r},\omega) = \mathbf{0} \tag{27}$$

Subject to the boundary conditions imposed by: (1) The boundary condition in the external surface of the cavity S_{out}:

$$\widehat{\mathbf{n}}_{\mathrm{out}} \times \mathbf{E}_2(\mathbf{r},\omega) = \mathbf{0} \tag{28}$$

(2) The continuity of the fields at S_{in}

$$\widehat{\mathbf{n}}_{\mathrm{in}} \times \mathbf{E}_2(\mathbf{r},\omega) = \widehat{\mathbf{n}}_{\mathrm{in}} \times \mathbf{E}_1(\mathbf{r},\omega) \tag{29}$$

$$\widehat{\mathbf{n}}_{\mathrm{in}} \times \mathbf{H}_2(\mathbf{r},\omega) = \widehat{\mathbf{n}}_{\mathrm{in}} \times \mathbf{H}_1(\mathbf{r},\omega) \tag{30}$$

$$\varepsilon_2(\omega)\,\widehat{\mathbf{n}}_{\mathrm{in}} \cdot \mathbf{E}_2(\mathbf{r},\omega) = \varepsilon_1(\omega)\,\widehat{\mathbf{n}}_{\mathrm{in}} \cdot \mathbf{E}_1(\mathbf{r},\omega) \tag{31}$$

$$\widehat{\mathbf{n}}_{\mathrm{in}} \cdot \mathbf{H}_2(\mathbf{r},\omega) = \widehat{\mathbf{n}}_{\mathrm{in}} \cdot \mathbf{H}_1(\mathbf{r},\omega) \tag{32}$$

We assume further that the substance homogeneously filling V_1 is characterized by a positive relative permittivity $\varepsilon_1(\omega) = \varepsilon_i > 0$, whose frequency-dispersive properties can be neglected over the bandwidth of interest. On the other hand, the substance filling V_2 is characterized by a dispersive permttivity that equals zero at the frequency ω_p, i.e., $\varepsilon_2(\omega = \omega_p) = 0$ (the plasma frequency or the epsilon-near-zero (ENZ) frequency). For instance, $\varepsilon_2(\omega)$ might

be characterized by a Drude frequency-dispersion profile: $\varepsilon_2(\omega) = 1 - \omega_p^2/\omega^2$. In this manner, at the frequency ω_p the system of wave equations (26)-(27) subject to the boundary conditions (28)-(32) reduces to

$$\nabla \times \nabla \times \mathbf{E}_2(\mathbf{r}, \omega_p) = \mathbf{0} \tag{33}$$

$$\nabla \times \nabla \times \mathbf{E}_1(\mathbf{r}, \omega_p) - \frac{\omega_p^2}{c^2}\,\varepsilon_i\,\mathbf{E}_1(\mathbf{r}, \omega_p) = \mathbf{0} \tag{34}$$

with

$$\widehat{\mathbf{n}}_{\rm out} \times \mathbf{E}_2(\mathbf{r}, \omega_p) = \mathbf{0} \tag{35}$$

$$\widehat{\mathbf{n}}_{\rm in} \times \mathbf{E}_2(\mathbf{r}, \omega_p) = \widehat{\mathbf{n}}_{\rm in} \times \mathbf{E}_1(\mathbf{r}, \omega_p) \tag{36}$$

$$\widehat{\mathbf{n}}_{\rm in} \times \mathbf{H}_2(\mathbf{r}, \omega_p) = \widehat{\mathbf{n}}_{\rm in} \times \mathbf{H}_1(\mathbf{r}, \omega_p) \tag{37}$$

$$\widehat{\mathbf{n}}_{\rm in} \cdot \mathbf{E}_1(\mathbf{r}, \omega_p) = \mathbf{0} \tag{38}$$

$$\widehat{\mathbf{n}}_{\rm in} \cdot \mathbf{H}_2(\mathbf{r}, \omega_p) = \widehat{\mathbf{n}}_{\rm in} \cdot \mathbf{H}_1(\mathbf{r}, \omega_p) \tag{39}$$

In this manner, if we find the geometries for which there is a solution to the above system of equations and boundary conditions, we can determine the set of cavities for which there is an eigenmode at the plasma frequency. In principle, there are many possible solutions. Here, we take advantage of the results of the previous section (i.e., the fact that ENZ media support spatially "electrostatic" field distributions) and focus on the set of eigenmodes that have spatially "electrostatic" field distributions in V_2. In other words, we can write the electric field distribution in V_2 as the gradient of a scalar potential, $\mathbf{E}_2(\mathbf{r}, \omega_p) = -\nabla\varphi_2(\mathbf{r})$, with $\nabla^2\varphi_2(\mathbf{r}) = 0$, accompained by a zero magnetic field $\mathbf{H}_2(\mathbf{r}, \omega_p) = \mathbf{0}$. Thus, the existence of an eigenmode in the cavity for which the field distribution in V_2 is spatially "electrostatic", is determined by the existence of solutions to the following system of differential equations:

$$\nabla^2\varphi_2(\mathbf{r}) = 0,\ \mathbf{E}_2(\mathbf{r}, \omega_p) = -\nabla\varphi_2(\mathbf{r}) \tag{40}$$

$$\nabla \times \nabla \times \mathbf{E}_1(\mathbf{r}, \omega_p) - \frac{\omega_p^2}{c^2}\,\varepsilon_i\,\mathbf{E}_1(\mathbf{r}, \omega_p) = \mathbf{0} \tag{41}$$

subject to the the boundary conditions

$$\widehat{\mathbf{n}}_{\rm out} \times \mathbf{E}_2(\mathbf{r}, \omega_p) = \mathbf{0} \tag{42}$$

$$\widehat{\mathbf{n}}_{\rm in} \times \mathbf{E}_2(\mathbf{r}, \omega_p) = \widehat{\mathbf{n}}_{\rm in} \times \mathbf{E}_1(\mathbf{r}, \omega_p) \tag{43}$$

$$\widehat{\mathbf{n}}_{\rm in} \times \mathbf{H}_1(\mathbf{r}, \omega_p) = \mathbf{0} \tag{44}$$

$$\widehat{\mathbf{n}}_{\rm in} \cdot \mathbf{E}_1(\mathbf{r}, \omega_p) = \mathbf{0} \tag{45}$$

$$\widehat{\mathbf{n}}_{\rm in} \cdot \mathbf{H}_1(\mathbf{r}, \omega_p) = \mathbf{0} \tag{46}$$

Let us inspect the conditions under which there is a solution for this system of differential equations/boundary conditions. First, (44)-(46) impose some restrictions on the internal particle. In particular, the particle must support a field distribution that has zero (normal and tangential) magnetic field, and zero normal electric field at its boundary. However, if we can find a particle with those properties, it will be shown later that a cavity composed by such particle and a ENZ host supports an eigenmode at the plasma frequency independently of the geometry of the ENZ host. Moreover, one can readily identify a few examples with canonical geometries. These include: (1) Homogeneous dielectric spheres with radii r_i so that $\widehat{J}_n(k_i r_i) = 0$ for $n = 1, 2, 3...$, (2) Homogeneous dielectric cylinders of arbitrary height h, but whose bottom and top faces are covered with perfect-magnetic-conducting (PMC) layers, and with radii r_i so that $J_n(k_i r_i) = 0$ for $n = 1, 2, 3...$ In principle, it should be possible to find other suitable particles via numerical optimization. Similarly, the same conditions could be satisfied in 2D systems, for example with infinitely long cylinders with radius r_i so that $J_n(k_i r_i) = 0$ for $n = 1, 2, 3...$

Therefore, let us assume that the cavity contains one of the aforementioned particles. Consequently, the overall cavity will support an eigenmode at ω_p as long as there is a solution to Laplace's equation $\nabla^2 \varphi_2 (\mathbf{r}) = 0$, with $\mathbf{E}_2 (\mathbf{r}, \omega_p) = -\nabla \varphi_2 (\mathbf{r})$, subject to the boundary conditions $\widehat{\mathbf{n}}_{\text{out}} \times \mathbf{E}_2 (\mathbf{r}, \omega_p) = \mathbf{0}$ and $\widehat{\mathbf{n}}_{\text{in}} \times \mathbf{E}_2 (\mathbf{r}, \omega_p) = \widehat{\mathbf{n}}_{\text{in}} \times \mathbf{E}_1 (\mathbf{r}, \omega_p)$, where $\widehat{\mathbf{n}}_{\text{in}} \times \mathbf{E}_1 (\mathbf{r}, \omega_p)$ is imposed by the internal particle. Note that specificying the tangential components of the electric field is equivalent to fix the tangential derivatives of the potential, and that this is equivalent to fixing the potential itself (see, e.g., [7] p. 36). This property appears commonly in electrostatic problems with dielectric bodies, where the continuity of the tangential components of the electric field at the interface between two dielectric bodies, $\widehat{\mathbf{n}} \times \mathbf{E}$, is expressed as the continuity of the potential itself φ [7]. Therefore, the eigenmode will exist if there is a solution to Laplace´s equation in V_2 subject to the Dirichlet boundary condition imposed by the external surface of the cavity, and the field distribution in the surface of the internal particle. Indeed, it is a classical result that the solution to the Dirichlet problem for Laplace's equation exists and is unique when the boundary is sufficiently smooth and the prescribed potential on the boundary is continuous (see, e.g., [8] p. 89). This ensures that there is a solution for almost any physically realizable cavity.

To finalize the proof, it can be readily checked that this solution, whatever it is, will not change the electromagnetic field prescribed on the surface of the dielectric partice. In particular, (1) The solution of the electrostatic problem is defined so that it preserves the tangential component of the electric field. (2) Even if the solution has a nonzero normal component of the electric field, $\widehat{\mathbf{n}}_{\text{in}} \cdot \mathbf{E}_2 (\mathbf{r}, \omega_p) \neq \mathbf{0}$, the normal component within the particle will continue to be zero, since $\varepsilon_2 (\omega_p) \widehat{\mathbf{n}}_{\text{in}} \cdot \mathbf{E}_2 (\mathbf{r}, \omega_p) = 0 = \varepsilon_i \widehat{\mathbf{n}}_{\text{in}} \cdot \mathbf{E}_1 (\mathbf{r}, \omega_p)$, with $\varepsilon_i \neq 0$. (3) By definition, this electrostatic field distribution since it is irrotational cannot generate any magnetic field on S_{in}.

In summary, it can be concluded that if we design a dielectric particle so that it enables the excitation of a spatially "electrostatic" field distribution in the ENZ host with the eigenfrequency the same as the plasma frequency of the ENZ host, then the cavity composed by the combination of this particle and ENZ host will always have an eigenmode at the plasma frequency, independently of the geometry of the ENZ host. Therefore, the cavity can be deformed resulting in significant changes of its shape, size and topology. As long as the geometry of the ENZ host supports a solution to the Dirichlet problem of Laplace's equation, the cavity will support an eigenmode at the plasma frequency. The theory has been numerically validated by using a commercial electromagnetic solver (see Methods). Specifically, we have included numerical examples of several ENZ cavities containing a dielectric sphere (Fig. 3 of the main text, and Supplementary Figures 11-18), as well as several cavities in which the internal particle consists of a dielectric cylinder whose top and bottom faces have been covered with PMC layers (Supplementary Figures 19-23).

Supplementary Note 4: Canonical Example: Core-shell spherical cavity

Specific examples help to understand the behavior of some of the modes present in cavities of arbitrary geometry. To this end, let us consider a cavity consisting of two concentric spheres, with internal and external radii of a and b, respectively, and with internal and external permittivities of ε_1 and ε_2, respectively (see Supplementary Figure 9). Due to its spherical symmetry, the modes excited in the cavity correspond to the spherical harmonics (12)-(15). The characteristic equations that determine the eigenfrequencies of each spherical harmonic can be found by imposing the boundary condition $\widehat{\mathbf{n}} \times \mathbf{E} = \mathbf{0}$ on the surface of the cavity, and enforcing the continuity of the fields on the surface between the two concentric spheres. This exercise leads to the following characteristic equations:

$$\frac{\widehat{Y}'_n(k_2 b)\widehat{J}'_n(k_2 a) - \widehat{J}'_n(k_2 b)\widehat{Y}'_n(k_2 a)}{\widehat{Y}'_n(k_2 b)\widehat{J}_n(k_2 a) - \widehat{J}'_n(k_2 b)\widehat{Y}_n(k_2 a)} = \frac{\eta_1}{\eta_2}\frac{\widehat{J}'_n(k_1 a)}{\widehat{J}_n(k_1 a)}, \quad \text{for } TM \text{ modes} \tag{47}$$

$$\frac{\widehat{Y}_n(k_2 b)\widehat{J}_n(k_2 a) - \widehat{J}_n(k_2 b)\widehat{Y}_n(k_2 a)}{\widehat{Y}_n(k_2 b)\widehat{J}'_n(k_2 a) - \widehat{J}_n(k_2 b)\widehat{Y}'_n(k_2 a)} = \frac{\eta_1}{\eta_2}\frac{\widehat{J}_n(k_1 a)}{\widehat{J}'_n(k_1 a)}, \quad \text{for } TE \text{ modes} \tag{48}$$

where η_1, k_1 and η_2, k_2 are the medium impedance and propagation constant of the internal and external regions, respectively. When the outer layer of the cavity is filled with a ENZ material ($\varepsilon_2 \to 0$), the characteristic equations can be asymptotically written as follows

$$\frac{\left(\frac{a}{b}\right)^{2n+1} - 1}{\frac{1}{n+1}\left(\frac{a}{b}\right)^{2n+1} + \frac{1}{n}} = \frac{\varepsilon_2}{\varepsilon_1}\,(k_1 a)\,\frac{\widehat{J}'_n(k_1 a)}{\widehat{J}_n(k_1 a)}, \quad \text{for } TM \text{ modes} \tag{49}$$

$$\frac{\left(\frac{a}{b}\right)^{2n+1} - 1}{(n+1)\left(\frac{a}{b}\right)^{2n+1} + n} = \frac{1}{k_1 a}\frac{\widehat{J}_n(k_1 a)}{\widehat{J}'_n(k_1 a)}, \quad \text{for } TE \text{ modes} \tag{50}$$

Note that the r.h.s. of (49) goes to zero since $\varepsilon_2 \to 0$, and hence, there is no solution for the TM modes except at $\widehat{J}_n(k_1 a) = 0$. This condition corresponds to a zero tangential magnetic field, which enables the existence of spatially electrostatic fields characterizing TM modes in the ENZ region, while at the same time satisfying the boundary conditions on the surface of the cavity.

Interestingly, when the volume of the internal sphere is much smaller than the volume of the whole cavity, $(a/b)^{2n+1} \ll 1$, the characteristic equations (49)-(50) can be approximated as follows

$$-n = \frac{\varepsilon_2}{\varepsilon_1}\,(k_1 a)\,\frac{\widehat{J}'_n(k_1 a)}{\widehat{J}_n(k_1 a)}, \quad \text{for } TM \text{ modes} \tag{51}$$

$$-\frac{1}{n} = \frac{1}{k_1 a}\frac{\widehat{J}_n(k_1 a)}{\widehat{J}'_n(k_1 a)}, \quad \text{for } TE \text{ modes} \tag{52}$$

Note that in this limit the characteristic equation becomes independent of the external surface even for the TE modes.

Supplementary Note 5: Perturbational Techniques

Here we use perturbational techniques (see, e.g., [6]) to ratify further that the eigenfrequencies of spatially electrostatic eigenmodes are preserved under geometrical transformations. To this end, let us consider a cavity of volume V enclosed within a surface S subject to the boundary condition $\widehat{\mathbf{n}} \times \mathbf{E} = \mathbf{0}$ (see Supplementary Figure 10). We assume that this cavity supports an eigenmode with fields $\mathbf{E}_0, \mathbf{H}_0$ at the eigenfrequecy ω_0. The cavity could be, for example, the spherical cavity described in the previous section. Next, we generate a second cavity by modifying the original cavity, leading to a volume $V' = V - \triangle V$ and $S' = S - \triangle S$. In general, this new cavity will support a different eigenmode with fields $\mathbf{E}, \mathbf{H}$ at an also different eigenfrequency ω. Note that the fields of the eigenmode in the first cavity satisfy time-harmonic curl Maxwell equations at frequency ω_0:

$$\nabla \times \mathbf{E}_0 = i\omega_0\mu_0\mathbf{H}_0 \tag{53}$$

$$\nabla \times \mathbf{H}_0 = -i\omega_0\varepsilon_0\varepsilon\mathbf{E}_0 \tag{54}$$

On the other hand, the fields of the eigenmode in the second cavity satisfy time-harmonic curl Maxwell equations at frequency ω:

$$\nabla \times \mathbf{E} = i\omega\mu_0\mathbf{H} \tag{55}$$

$$\nabla \times \mathbf{H} = -i\omega\varepsilon_0\varepsilon\mathbf{E} \tag{56}$$

In this manner, we can then write:

$$\nabla \cdot (\mathbf{H} \times \mathbf{E}_0^*) = \mathbf{H} \cdot \nabla \times \mathbf{E}_0^* - \mathbf{E}_0^* \cdot \nabla \times \mathbf{H} = -i\omega_0\mu_0\mathbf{H} \cdot \mathbf{H}_0^* + i\omega\varepsilon_0\varepsilon\mathbf{E}_0^* \cdot \mathbf{E} \tag{57}$$

$$\nabla \cdot (\mathbf{H}_0^* \times \mathbf{E}) = \mathbf{H}_0^* \cdot \nabla \times \mathbf{E} - \mathbf{E} \cdot \nabla \times \mathbf{H}_0^* = i\omega\mu_0\mathbf{H}_0^* \cdot \mathbf{H} - i\omega_0\varepsilon_0\varepsilon\mathbf{E} \cdot \mathbf{E}_0^* \tag{58}$$

Next, integrating over V', and applying the divergence theorem we get

$$\oint_{S'} (\mathbf{H} \times \mathbf{E}_0^* + \mathbf{H}_0^* \times \mathbf{E}) \cdot \widehat{\mathbf{n}} dS' = i(\omega - \omega_0) \int_{V'} (\mu_0\mathbf{H}_0^* \cdot \mathbf{H} + \varepsilon_0\varepsilon\mathbf{E}_0^* \cdot \mathbf{E})\, dV' \tag{59}$$

However, since $\widehat{\mathbf{n}} \times \mathbf{E} = \mathbf{0}$ on S' we have

$$\oint_{S'} (\mathbf{H} \times \mathbf{E}_0^* + \mathbf{H}_0^* \times \mathbf{E}) \cdot \widehat{\mathbf{n}} dS' = \oint_{S'} (\mathbf{H} \times \mathbf{E}_0^*) \cdot \widehat{\mathbf{n}} dS' \tag{60}$$

Moreover, since $\widehat{\mathbf{n}} \times \mathbf{E}_0 = \mathbf{0}$ on S and $S' = S - \Delta S$ we get

$$\oint_{S'} (\mathbf{H} \times \mathbf{E}_0^*) \cdot \widehat{\mathbf{n}} dS' = -\oint_{\triangle S} (\mathbf{H} \times \mathbf{E}_0^*) \cdot \widehat{\mathbf{n}} dS' \tag{61}$$

Therefore, the shift in the resonance frequency is given by

$$\omega - \omega_0 = \frac{i \oint_{\triangle S} (\mathbf{H} \times \mathbf{E}_0^*) \cdot \widehat{\mathbf{n}} dS'}{\int_{V'} (\mu_0\mathbf{H}_0^* \cdot \mathbf{H} + \varepsilon_0\varepsilon\mathbf{E}_0^* \cdot \mathbf{E})\, dV'} \tag{62}$$

We emphasize that (62) is not an approximation. It is a exact expression of the frequency shift due to the deformation. At the same time, (62) can only be evaluated with a complete knowledge of the eigenmode fields $\mathbf{E}_0$, $\mathbf{H}_0$, $\mathbf{E}$, $\mathbf{H}$. In general, one applies assumptions on the form of those fields in order to estimate the frequency shift. For example, a common approximation is to replace the perturbed eigenmode fields $\mathbf{E}, \mathbf{H}$ by the unperturbed eigenmode fields $\mathbf{E} \to \mathbf{E}_0$, $\mathbf{H} \to \mathbf{H}_0$, which is a reasonable estimate for small deformations [6]. In our case, since the magnetic field of the unperturbed mode in the ENZ region is zero $\mathbf{H}_0 = 0$, it follows from (62) that the approximate substitution $\mathbf{H} \to \mathbf{H}_0$ results in a zero frequency shift, even if the electric field in the volume ΔV is significanly large.

Supplementary References

[1] COMSOL Multiphysics (www.comsol.com).

[2] M. J. Weber, Handbook of optical materials (2003).

[3] W. G. Spitzer, D. Kleinman, D. Walsh, Phys. Rev. 113, 127–132 (1959).

[4] R. E. Collin, Field Theory of Guided Waves (IEEE Press, New York, 1991), 2nd ed.

[5] M. Silveirinha and N. Engheta, Phys. Rev. B 75, 075119 (2007).

[6] R. F. Harrington, Time-Harmonic Electromagnetic Fields (McGraw-Hill, New York, 1961).

[7] Landau, L. D., E. M. Lifshitz, and L. P. Pitaevskii. "Electromagnetics of continuous media." Pergamon Press, Oxford 1984.

[8] John, F. “Partial Differential Equations”. 3rd edition. Applied Mathematical Sciences 1, Springer-Verlag, New York 1978.
